# Investigation of switching uniformity in resistive memory via finite element simulation of conductive-filament formation

**DOI:** 10.1038/s41598-021-81896-z

**Published:** 2021-01-28

**Authors:** Kyunghwan Min, Dongmyung Jung, Yongwoo Kwon

**Affiliations:** Department of Materials Science and Engineering, Hongik University, Seoul, 04066 Korea

**Keywords:** Information storage, Electronic devices, Computational methods

## Abstract

Herein, we present simulations of conductive filament formation in resistive random-access memory using a finite element solver. We consider the switching material, which is typically an oxide, as a two-phase material comprising low- and high-resistance phases. The low-resistance phase corresponds to a defective and conducting region with a high anion vacancy concentration, whereas the high-resistance phase corresponds to a non-defective and insulating region with a low anion-vacancy concentration. We adopt a phase variable corresponding to 0 and 1 in the insulating and conducting phases, respectively, and we change the phase variable suitably when new defects are introduced during voltage ramp-up for forming. Initially, some defects are embedded in the switching material. When the applied voltage is ramped up, the phase variable changes from 0 to 1 at locations wherein the electric field exceeds a critical value, which corresponds to the introduction of new defects via vacancy generation. The applied voltage at which the defects percolate to form a filament is considered as the forming voltage. Here, we study the forming-voltage uniformity using simulations, and we find that for typical planar-electrode devices, the forming voltage varies significantly owing to the stochastic location of the initial defects at which the electric field is “crowded.” On the other hand, a protruding electrode can improve the switching uniformity drastically via facilitating the deterministic location of electric-field crowding, which also supported by the reported experimental results.

## Introduction

Resistive random-access memory (ReRAM) is an emerging nonvolatile memory (NVM) technology that offers the benefits of low operating voltage (of a few volts), high operating speed (nanoseconds to microseconds), and excellent cycling endurance (> 10^8^)^1–4^. ReRAM is considered a promising mainstream next-generation technology owing to its simple capacitor-like metal–insulator–metal (MIM) structure, which enables it to be further scaled down; moreover, it offers compatibility with the conventional complementary metal–oxide–semiconductor (CMOS) process. In addition, ReRAM devices are expected to find use in next-generation computing technologies such as neuromorphic computing and in-memory computing^5,6^. Some novel ReRAM materials other than metal oxides are also being investigated from the perspectives of improved device performance and eco-friendliness: organohalide perovskites have been reported to afford low switching voltages and high on/off ratios^7,8^. Moreover, biomemristors, which are based on natural biomaterials, can be implanted in the human body for human–machine interactions^9^.

Flash memory, which is the current mainstream NVM technology, is charge-based because its binary data depend on whether charges (mainly electrons) are stored in a floating gate. On the other hand, the binary data in ReRAM are determined by the state of the switching material: insulating or conductive. The stability, that is, data retention of ReRAM over flash memory can be understood by means of the following example: A 20-nm planar flash memory device has only ~ 100 electrons in its floating gate^10,11^. The stored electrons gradually leak, which leads to eventual data retention failure. On the other hand, a 20-nm metal-oxide ReRAM contains ~ 100,000 atoms in a single storage node (20 nm^3^). It is difficult for atoms to escape spontaneously from the storage node relative to electrons, which implies that ReRAM devices can exhibit superior data retention over flash memory for the same memory size.

The formation of a conductive filament (CF) is the key aspect underlying the switching mechanism of ReRAM devices. As mentioned earlier, a ReRAM cell comprises an insulator layer sandwiched between two metal electrodes, *i.e.*, the MIM structure. The insulator is in the insulating state by default. This state is called the high-resistance state (HRS). When a certain voltage is applied to the HRS cell, a CF is formed between the top and bottom electrodes, which switches the cell to the low-resistance state (LRS). This initial formation of a CF is called “forming,” and the corresponding applied voltage is called the forming voltage, ${V}_{form}$. The LRS switches back to the HRS upon the rupture of the CF, which is called the reset operation. Here, we note that a reverse bias is used for the reset in the case of bipolar ReRAMs. The ruptured CF can be reconnected by applying a forward bias, which corresponds to the set operation. Once an initial CF is formed, reset and set operations can be performed repeatedly. Here, we note that the formation of CFs has been directly observed experimentally^12–15^.

The CF can comprise either anion vacancies or metal ions^16^. The former corresponds to the valence change mechanism (VCM), and the latter corresponds to electrochemical metallization (ECM). In general, noble-metal electrodes such as those of gold and platinum afford VCM, which results in valence change RAM (VC-RAM), whereas reactive-metal electrodes such as silver and copper afford ECM, which results in conductive bridge RAM (CB-RAM), although the detailed mechanism differs for the constituent insulator and electrode materials.

Hereafter, we focus on VC-RAM, wherein anion vacancies are generated when a sufficiently strong electric field is applied. A CF is formed when the vacancies form a percolation path, *i.e.*, a soft dielectric breakdown^17^. This process has a random nature, as is evident from the wide distribution of the forming voltage reported. This large randomness necessarily leads to nonuniformities of the forming voltage, which pose a major challenge to the design of large-capacity memories having or exceeding the order of gigabytes. Therefore, it is very important to study this nonuniformity during switching and determine solutions to reduce it. In this regard, many efforts have been expended to improve the switching uniformity^18–24^.

Meanwhile, device simulation has played a very important role in the research and development of modern logic CMOS and memory products. For commercial silicon devices, a standardized device simulation tool called technology computer-aided design (TCAD) is widely used; chip makers employ TCAD during the early stages of development to predict the device performance (usually, on-current) according to the architecture (device geometry and constituent materials) and the applied biases. In other words, TCAD plays an important role in estimating the optimal combination of device geometry and materials. In this context, it is evident that the device model for ReRAM must be ready before ReRAM-device fabrication progresses to the industrial development stage.

Current ReRAM-device simulations include the random circuit breaker (RCB) model^25^, hybrid simulations based on particle dynamics (PD) and the finite element method (FEM)^26,27^, and FEM simulations with commercial tools^28–32^. The most advanced model, which is now commercialized^33^, is presented in Refs.^26^ and^27^, using which the formation and breakdown of CFs can be visualized by tracking the formation and migration of oxygen ions and vacancies according to the electric field and temperature gradient for a 5-nm-thick HfO_2_ ReRAM. However, this model may require very large computing resources. Meanwhile, the TCAD-based model presented in Refs.^28^ and^29^ also analyzes the switching behavior by calculating the probability of defect formation and extinction at the macroscale level. This model facilitates observations of the distribution of the forming voltage. However, in this model, the shape of the formed CF is not fully expressed because the actual radius of the defect is not defined. Instead, a defect is assigned at a volume element, which means that the mesh size is the defect size. On the other hand, in FEM simulations using commercial multiphysics software^30–32^, the switching behavior has been analyzed for a preformed CF, which does not model the initial forming-process.

Currently, FEM is far more effective for device simulations than molecular dynamics (MD). Although MD simulations require massive computing resources (large-scale hardware and lengthy CPU times), they only afford calculations at nanometer-lengths and picosecond-time scales. Accordingly, it is difficult to calculate the full resistive switching, a phenomenon whose length and time scales are up to ~ 100 nm and microseconds, respectively. Therefore, the MD simulations are inadequate for the investigation of device architectures including electrodes and other components; however, MD simulations are sufficiently powerful to reveal some basic switching mechanisms.

From this standpoint, we herein present an FEM simulation that can reproduce the stochasticity of CF formation. For a given device structure with material properties and boundary conditions (voltage, temperature), the model calculates the local electric field, probability of defect formation, and stochastic CF formation. The material parameters include the defect formation energy, bond polarization factor, defect radius, and electrical conductivity of the HRS and LRS. This model allows us to observe the difference in the CF formation depending upon the parameter values and device structure; furthermore, we can estimate the nonuniformity in the forming voltage due to the stochasticity. Most importantly, FEM models that do not require high-performance computing are the most efficient for testing various device architectures; thus, FEM simulations are extremely important to conduct prior to novel devices progressing to the mass-production stage. Here, using this model, we further demonstrate that a protruding electrode can improve the switching uniformity due to electric-field “crowding” at the protruding tip.

## Simulation procedure development

**Figure 1:**
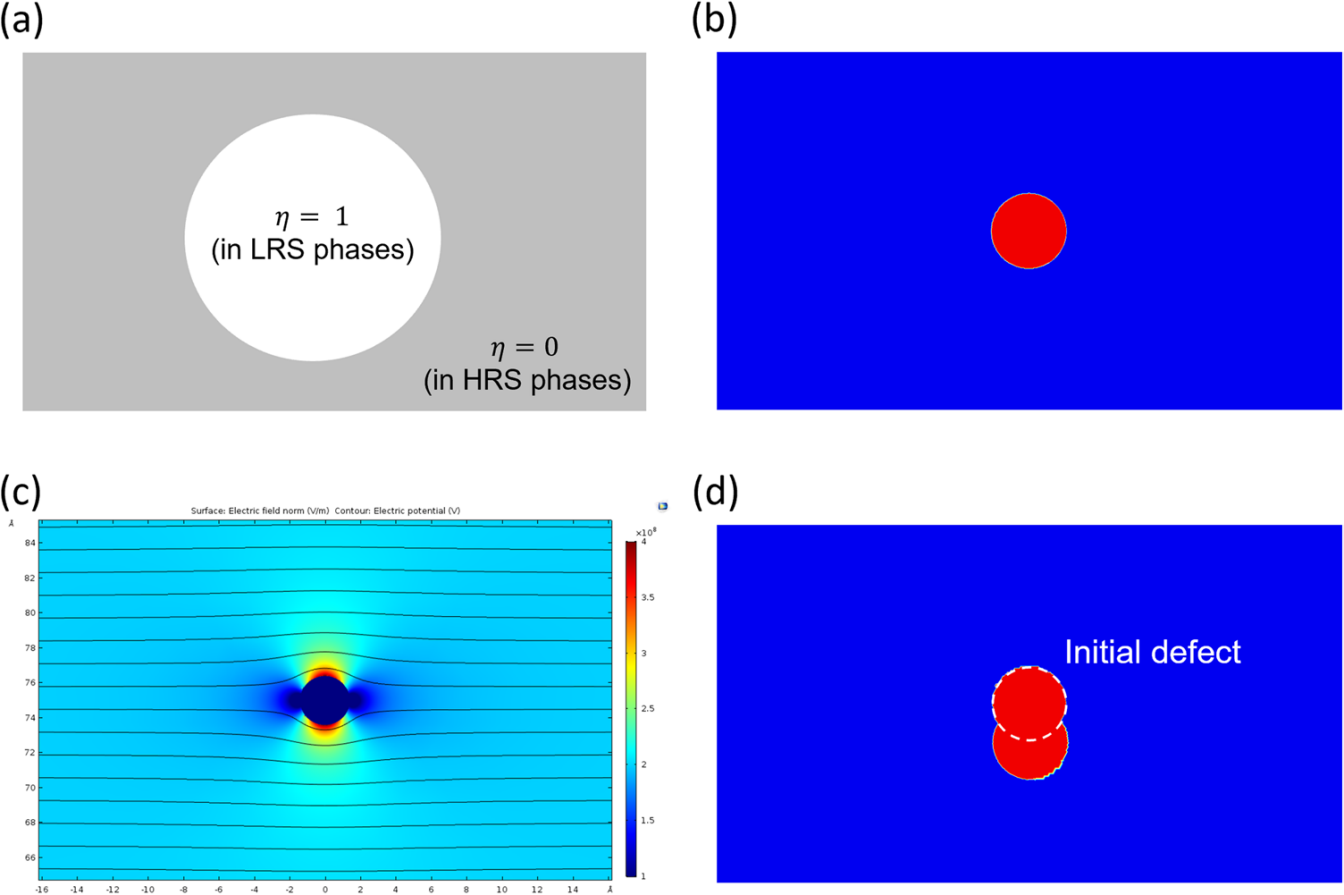
Representation of our simulation system and electric-field-dependent defect generation: (**a**) value assignment to phase variable ${\upeta }\left( {\vec{r}} \right)$ for low- and high-resistance phases (LRPs and HRPs, respectively), (**b**) a defect LRP embedded in an HRP matrix, (**c**) electric-field distribution (colored) with equipotential lines, and (**d**) new defect generated below the initial defect region.

We simulated a forming process based on the stochastic arrangement of initial defects (small LRS regions) and the electric-field-dependent defect-generation rate. Here, we remark that it is not possible to explicitly track individual defects in the FEM simulation. Instead, we regard the switching material as a two-phase mixture comprising low- and high-resistance phases (LRPs and HRPs). The LRP and HRP correspond to the defective and non-defective phases, respectively; in other words, a CF consists of LRPs. In our approach, we adopt a phase variable, $\upeta (\overrightarrow{r},\mathrm{t})$, to represent the two-phase material, as shown in Fig. 1a.

1 $$ {\upeta }\left( {\vec{r},{\text{t}}} \right) = \left\{ {\begin{array}{*{20}c} {0{\text{, at a position within an HRP}}} \\ {1{\text{, at a position within an LRP}}} \\ \end{array} } \right. $$

Here, $\vec{r}$ denotes the position vector and ${\text{t}}$ denotes the time. The phase variable has binary values of 0 and 1 within the phases. The adoption of the phase variable was benchmarked from the phase-field method that is commonly used to predict the microstructure evolution in materials science^34^.

The simulation geometry is the MIM structure of the device. A positive bias, $V\left( t \right)$, is applied to the top electrode, while the bottom electrode is grounded. The simulation begins from $V\left( {t = 0} \right) = 0$ V. The applied voltage increases over time at a fixed ramp-up rate such that $V\left( t \right) = at$, where $a$ denotes the ramp-up rate [$V/s$]. The evolution of the phase variable with the voltage ramp-up corresponds to CF formation.

Let us consider an MIM structure wherein a circular LRP is embedded in the HRP matrix. Figure 1b shows the vicinity of such an LRP. First, the electric potential distribution is obtained for this system when a voltage is applied to the top electrode. Upon charge neutrality being assumed at all local positions, we can express the current continuity equation as below:

2 $$ \nabla \cdot \vec{J} + \frac{\partial \rho }{{\partial t}} = 0, $$

where $\vec{J}$ denotes the current density, and $\rho$ denotes the total charge density. Under the charge neutrality assumption, the second term reduces to 0 ($\rho = 0$). We know that $\vec{J} = - \,\sigma \nabla \phi$, where $\sigma$ denotes the electrical conductivity, $\phi$ denotes the electric potential, and $- \nabla \phi$ denotes the electric field; consequently, Eq. (2) becomes

3 $$ \nabla \cdot \left[ {\sigma \nabla \phi } \right] = 0 $$

Solving Eq. (3) yields the electric potential distribution, $\phi$, electric field distribution, $\vec{E} = - \nabla \phi$, and current density distribution, $\vec{J} = \sigma \vec{E}$, in turn. Here, we note that the electrical conductivity is also a function of position and time, and it evolves along with the phase variable as below:

4 $$ \sigma \left( {\vec{r},{\text{t}}} \right) = \sigma_{H} + \sigma_{L} {\upeta }\left( {\vec{r},{\text{t}}} \right) $$

Here, $\sigma_{H}$ and $\sigma_{L}$ represent constant material parameters corresponding to the conductivities of the HRP and LRP, respectively. We also note that the current density $\vec{J}$, electric potential $\phi$, and electric field $\vec{E}$ are both position- and time-dependent.

Next, we obtain the defect-generation rate, $G\left( {\vec{r},{\text{t}}} \right)$, using the electric field^26,27^.

5 $$ G = G_{0} \exp \left[ { - \frac{{E_{a} - bE}}{{k_{B} T}}} \right]\sigma , $$

where $G_{0}$ denotes a pre-exponential factor [$cm^{ - 3} s^{ - 1}$], $E_{a}$ denotes the activation energy for defect formation [$eV$], $b$ denotes the bond polarization factor [$e \cdot cm]$, $k_{B}$ denotes the Boltzmann constant [$eV/K$], and $T$ denotes the absolute temperature. In this work, the temperature is fixed at 300 K because it is known that the Joule heating effect is negligible during the forming process^26,27^. Next, we calculate the Poisson probability, $P_{c} \left( {\vec{r},{\text{t}}} \right)$, for defect generation using $G\left( {\vec{r},{\text{t}}} \right)$ ^35–37^.

6 $$ P_{c} = 1 - \exp \left[ { - GV_{e} \Delta t} \right]{\upsigma } $$

Here, $\Delta t$ denotes the time-step and $V_{e}$ denotes the volume of each element in the mesh. We note here that individual meshes have different sizes; thus $V_{e}$ is position-dependent. In addition, we can set the adaptive mesh option to “on” during our simulation because the shape of the phases varies with time and the positions that “need” coarser and finer meshes also vary with time. In this case, $V_{e}$ is also a function of both position and time.

Next, random numbers between 0 and 1 are generated at all node positions in the mesh. We denote this random number array as $P\left( {\vec{r},t} \right)$. Subsequently, all the positions at which $P < P_{c}$ are estimated, and new spherical LRPs are assigned at these positions. As in Fig. 1c, we can clearly observe strong electric field regions above and below the existing LRP, and as in Fig. 1d, a new LRP is stochastically assigned below the existing LRP. Hereafter, a new small circular LRP is simply called a “defect.” The last step conducted at this time step is the calculation of the current for the applied voltage, $I = I\left( V \right)$, via the integration of the current density along the top or bottom surface of the active material. Thus, we have

7 $$ I = \mathop \smallint \limits_{S}^{{}} \vec{J} \cdot \hat{n}dS, $$

where ${\text{S}}$ denotes the top or bottom surface of the active material and $\hat{n}$ denotes the surface normal. The CF formation is simulated by repeating all these procedures at the next voltage steps. Finally, the simulation is stopped when the current value attains a compliance value that is set as a simulation parameter.

**Figure 2:**
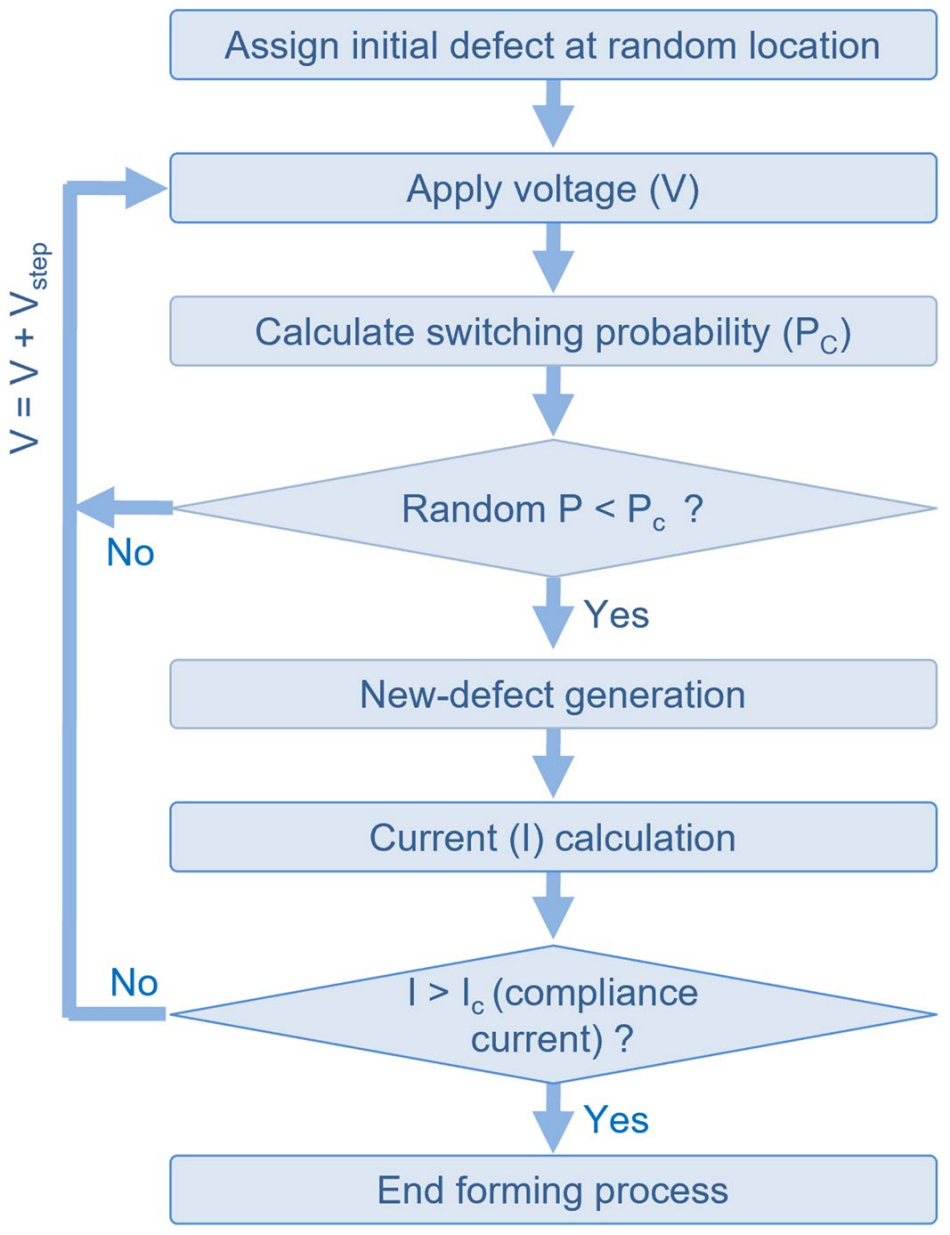
Flowchart for resistive random-access memory (ReRAM) forming simulation.

In this study, we used a commercial FEM solver, COMSOL Multiphysics^38^. A simulation example file for one initial defect at a random location, that, is a 1,138-line m-file for MATLAB is provided as the Supplementary Material. For the simulation, the main finite element package, COMSOL Multiphysics v5.3a, and two add-on modules, Electric Current (EC) and LiveLink for MATLAB, must be installed on a computer. This example simulation takes ~ 15 min to run on a Dell Precision T5810 workstation with an E5-1650 v4 processor and 32 GB of memory. The implementation of all the simulation procedures and the control of all simulation variables are conducted by the m-file; more details are available in the documentation for the COMSOL LiveLink for MATLAB. We also mention that the m-file may need to be modified for newer versions of COMSOL Multiphysics. All the aforementioned procedures are summarized in the flowchart shown in Fig. 2.

**Figure 3:**
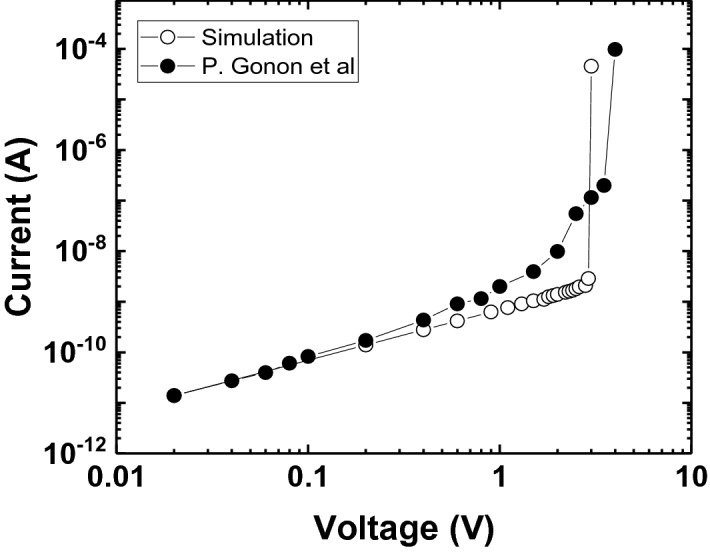
Comparison of simulated and experimental current–voltage curves for a 10-nm-thick HfO_2_ film. This simulation was carried out in 3D, and the experimental data were obtained from Gonon et al^41^.

Table 1 lists the simulation parameters considered in the study. We adopted hafnium oxide (HfO_2_), one of the most popular ReRAM materials, as the switching material. The defect formation energy and bond polarization factor of this material were obtained from the literature^39,40^; these two parameters most significantly influence the forming voltage. The defect radius was chosen to be close to the oxygen vacancy radius, and the conductivities were chosen to have a seven-order difference because larger differences result in numerical instability. In fact, the defect radius and conductivities are not critical for the forming voltage; the dependence of the forming voltage on certain parameters is discussed in the results and discussion section. Meanwhile, in the table, area factor $A_{f}$ denotes the width of the device along the perpendicular direction to the page, which is necessary for the calculation of current values. We remark here that the extension of this simulation to a 3D simulation is simple, although the 3D simulation can take several hours to run. For a 10-nm-thick HfO_2_ film, the switching voltage value from our 3D simulation agrees with that from an experiment^41^, as shown in Fig. 3. In fact, the $\sigma_{HRS}$_-_value in Table 1 was calibrated to the experimental current values for low voltage values. The difference in current at high voltage over ~ 0.5 V is because our simulation does not consider high field effects such as space-charge limited current.

**Table 1:** Simulation parameters.

Parameter	Value
Defect formation energy ($E_{a}$)^29^	5.9 eV
Bond polarization factor ($b$)^30^	91.8 eÅ
Electrical conductivity of defect ($\sigma_{LRS}$)	3.5 $\times { }$ 10^4^ S/m
Electrical conductivity of oxide ($\sigma_{HRS}$)	3.0 $\times$ 10^–3^ S/m
Defect radius ($R$)	1.4 Å
Computational domain size (2D)	
Width	50 nm
Thickness	5 nm, 10 nm, 15 nm
Area factor ($A_{f}$)	50 nm

## Results and discussion

### Defect-free system

**Figure 4:**
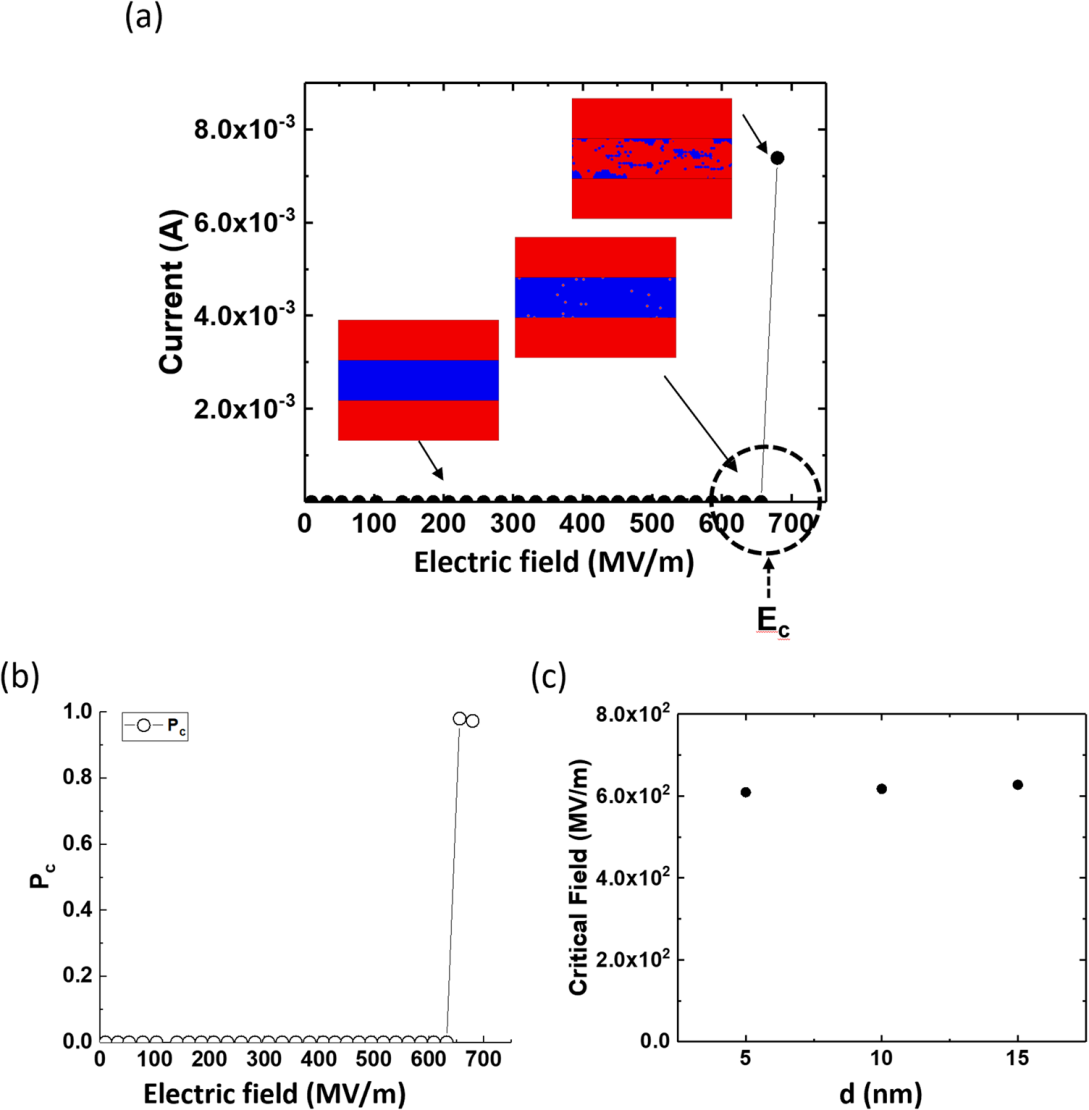
Simulation results for defect-free systems with no interface roughness and no initial defects: (**a**) current and (**b**) defect-generation probability as functions of the applied electric field for 5-nm-thick HfO_2_ device, and (**c**) critical electric field as a function of the HfO_2_ thickness. Insets in (**a**) show the phase distributions at three applied electric fields. The red region denotes the LRP that includes the top and bottom electrodes, and dark blue denotes the HRP.

We first performed simulations on ideal MIM structures with no defect (no initial LRP) in the switching material and no interface roughness. In this case, no new defect is generated until the magnitude of the electric field reaches a certain critical value, $E_{C}$. Once the electric field reaches $E_{C}$, new defects suddenly appear across the entire switching material, as shown in Fig. 4a. The $P_{C}$ value drastically increases at $E_{C}$, as shown in Fig. 4b, and in this case, the uniform electric field yields a uniform probability. Therefore, nearly the entire region transforms into an LRP. Thus, we can conclude that a defect-free MIM structure with a perfect interface cannot produce a CF. In actual ReRAM devices, it is thought that some roughness at the interfaces and that initial defects are present within the system. Such inhomogeneity results in electric-field crowding at a certain location, where a CF starts to grow. In this regard, Vandelli et al. mentioned that pre-existing defects are likely present at grain boundaries (GBs), where anion vacancies are more easily generated^26,42^. Furthermore, the $E_{C}$ value for the ideal MIM structure does not change although the insulator film thickness changes, as shown in Fig. 4c.

### Dependence of initial defect position

In an inhomogeneous rectangular system comprising LRP and HRP regions, the electric field is not uniform when biases are applied between the top and bottom electrodes. In the case of a circular LRP inclusion in the HRP matrix, the electric field is concentrated above and below the LRP inclusion as shown in Fig. 1c. Accordingly, new LRPs are more likely to appear above and/or below the initial LRP, as shown in Fig. 1d. During the forming process, the temperature within the device is maintained at 300 K because a current path is not formed, and thus, there is no occurrence of Joule heating arising from high current density^26,27^. At 300 K, the drift velocity of oxygen vacancies is less than ~ 1 pm/s at the high electric field of ~ 1,00 MV/m^31^. Hence, we do not include either thermal or vacancy drift effects in our forming simulation. Those must be included for reset and set simulations which are our future work.

**Figure 5:**
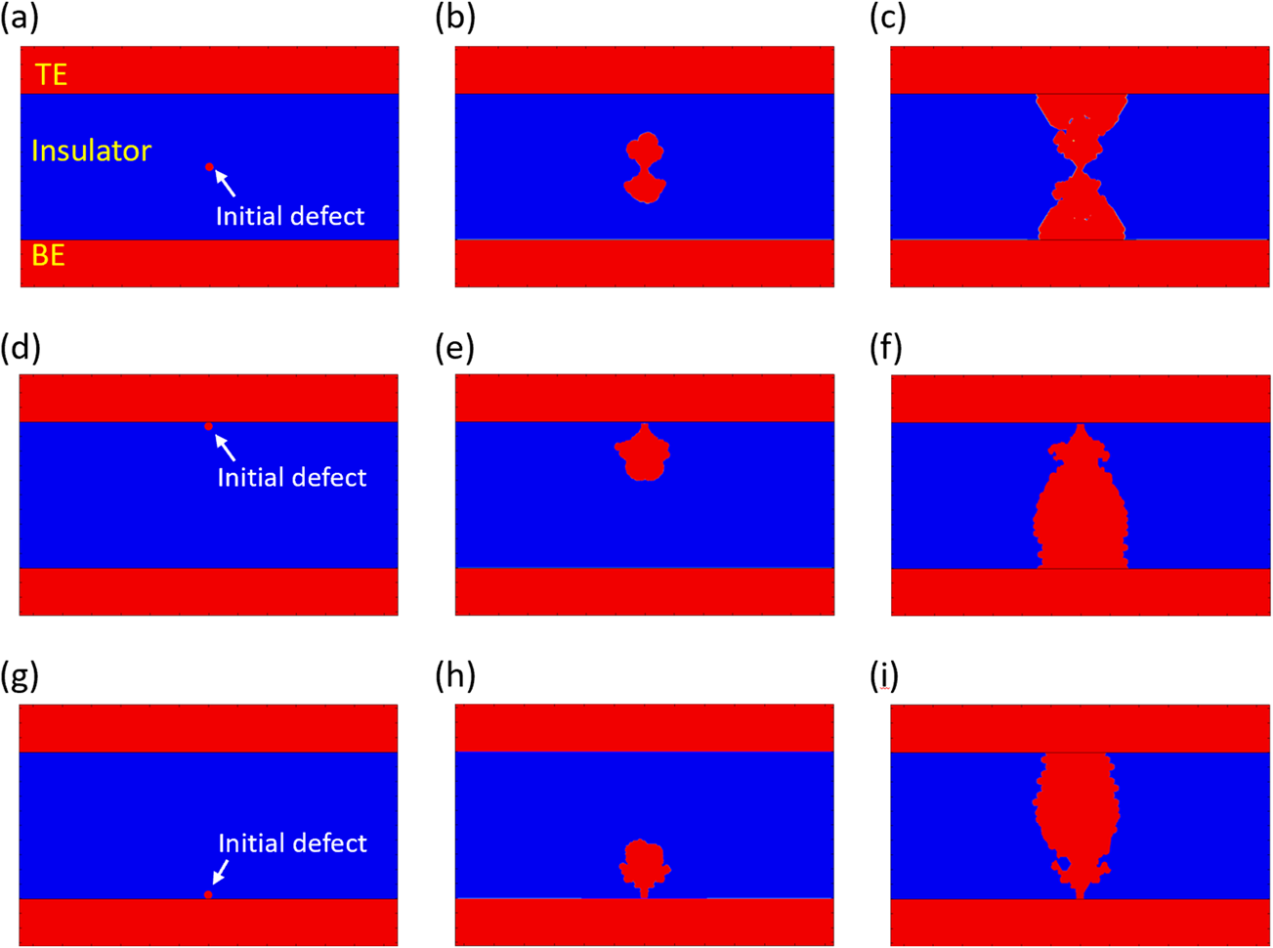
Evolution of conductive filament during voltage ramp-up when the initial defect is (**a**–**c**) at the middle position, (**d**–**f**) adjacent to the top electrode, and (**g**–**i**) adjacent to the bottom electrode. TE denotes the top electrode and BE denotes the bottom electrode. The insulator thickness is 5 nm.

In the study, we first simulated a system with an initial defect. Here, we note that while multiple defects may exist, there must exist at least one defect around which the electric field is maximally concentrated. Therefore, the initial defect can be considered as the site at which the electric field becomes the strongest. Figure 5 shows the different shapes of the resulting CFs depending on the position of the initial defect. The initial defect at the middle position leads to the formation of an hourglass-shaped CF, as shown in Fig. 5a–c. On the other hand, initial defects close to the electrodes afford roughly triangular CFs, as shown in Fig. 5d–i. In all cases, the CF starts to grow from the initial defect where the electric field is crowded.

**Figure 6:**
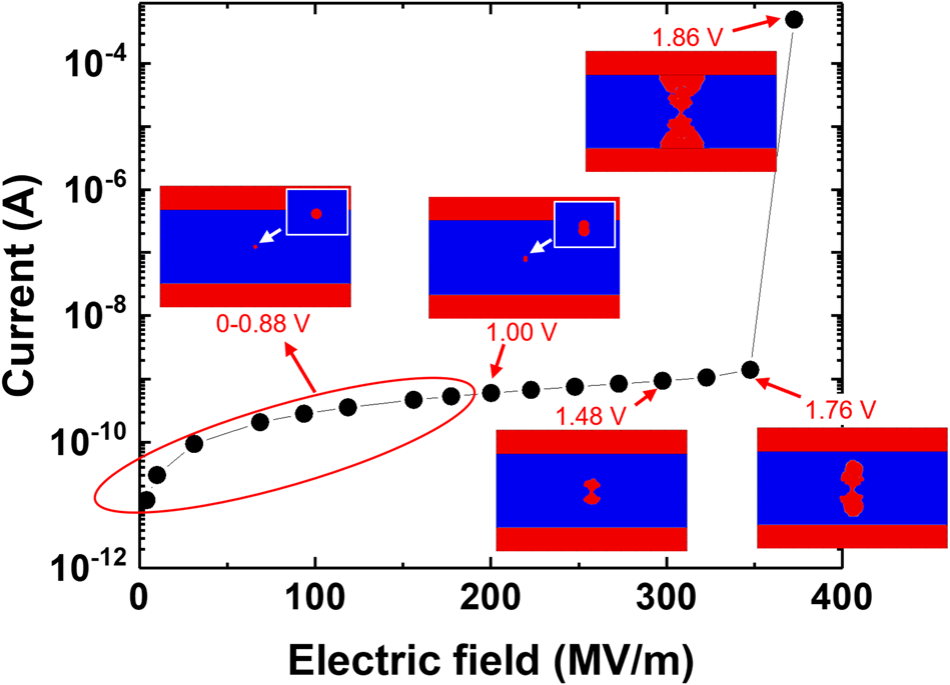
Switching curve for the case when the initial defect is at the middle position, which corresponds to Fig. 4a–c. The evolution of the conductive filament is shown at selected voltages. The initial defect does not change up to 0.88 V, and another defect is generated at 1 V (insets). The insulator thickness is 5 nm.

The current–voltage curve for a 2D MIM with one defect is presented in Fig. 6. We note that there is no drastic change in the current until the applied voltage reaches 1.48 V. In other words, no new defects are observed in the lower voltage regime because there is no mesh point wherein the local electric field reaches the critical field, $E_{C}$. A CF starts to grow rapidly from 1.48 V onward, and the current value increases by more than five orders at 1.86 V, which we consider as the forming voltage, $V_{form}$. The simulation is stopped when the CF connects the top and bottom electrodes. Here, we note that there may be multiple initial defects. In particular, we point out that a CF is likely to form at GBs, where the defect formation energy is lower^42^. The GB can be regarded as a serial arrangement of initial defects. While this situation can be currently modeled with our simulation, we plan to account for the GB effects in our future studies.

### Dependence of material parameters

**Figure 7:**
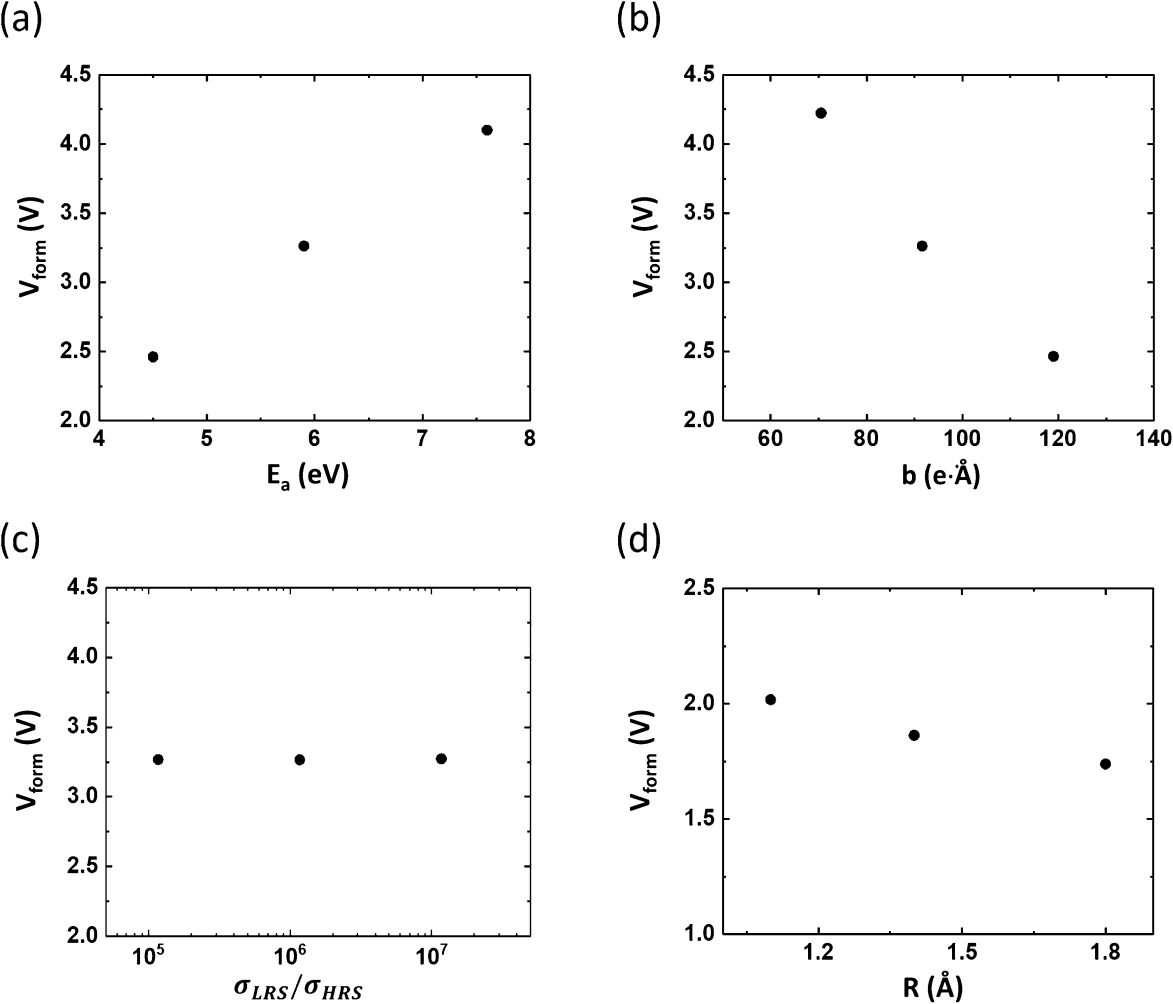
Material-parameter-dependence of forming voltage. (**a**) Defect formation energy, $E_{a}$, (**b**) bond polarization factor, $b$, (**c**) ratio of low-resistance state (LRS)-to-high-resistance state (HRS) conductivities, $\sigma_{LRS} /\sigma_{HRS}$, and (**d**) defect radius, $R$. The insulator thickness for these simulations is 10 nm.

Figure 7 shows the variation in $V_{form}$ with the material parameters of interest. We note that the influential parameters are the activation energy for defect formation, $E_{a}$, and the bond polarization factor, $b$. Forming voltage $V_{form}$ decreases with decreasing $E_{a}$ and increasing $b$, as shown in Fig. 7a,b, respectively, which can be clearly deduced from the defect-generation rate given by Eq. (5). In addition, there is no dependence on the conductivity difference between the HRP and LRP (Fig. 7c). In fact, the conductivity difference does not affect the electric-field distribution. Moreover, a larger defect size affords only a slight decrease in $V_{form}$, as shown in Fig. 7d, because a larger defect size results in higher electric-field crowding owing to the decreased distance for voltage drop—that is, the film thickness minus the defect size.

### Dependence of device geometry

**Figure 8:**
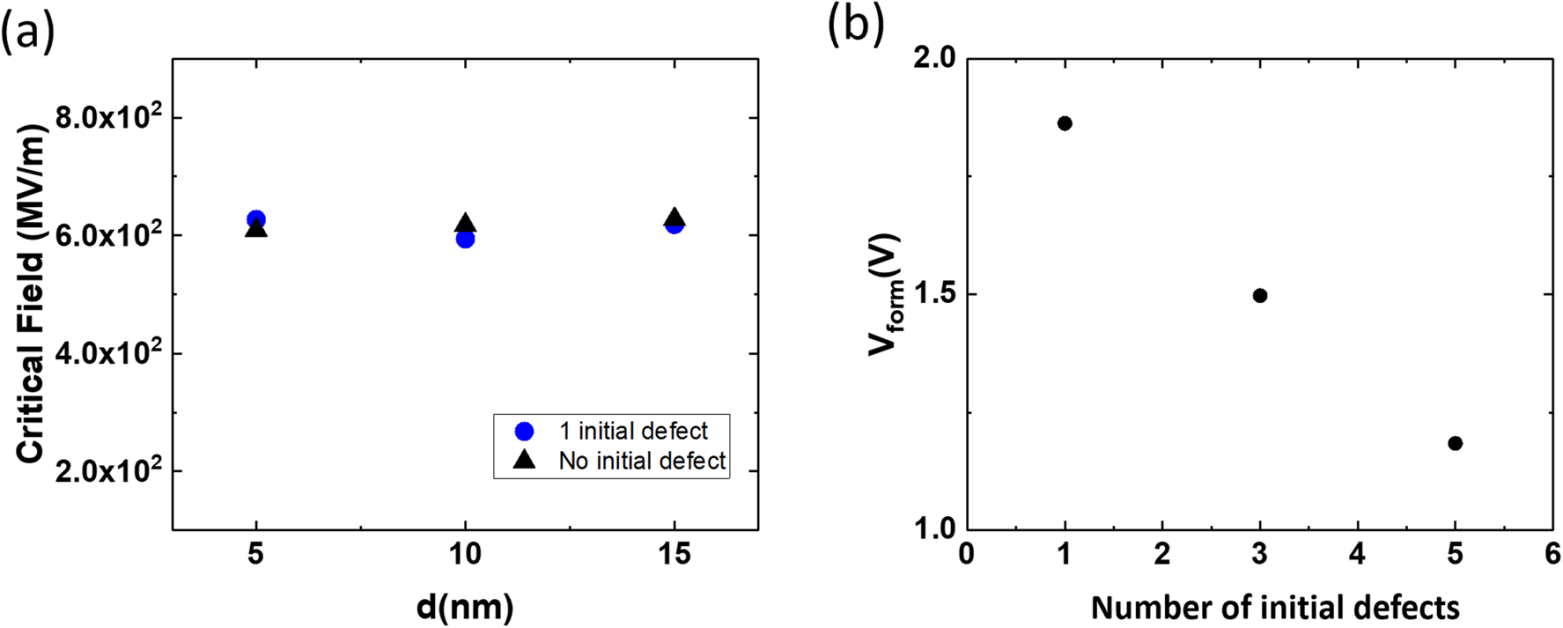
(**a**) Film thickness vs. critical field (maximum local electric field) when new defects are generated. (**b**) Number of initial defects vs. forming voltage when the initial defects are vertically and evenly distributed. The insulator thickness is 5 nm.

The configuration of initial defects greatly affects $V_{form}$. Here, we recall the concept of the critical field, $E_{C}$, for defect generation for the defect-free system. Defect generation at a specific location is determined by whether the “local electric field” exceeds the $E_{C}$ value. The $E_{C}$ values are the same regardless of the existence of the initial defect, as shown in Fig. 8a, although $V_{form}$ decreases with an increase in the number of initial defects, as shown in Fig. 8b, corresponding to the case wherein multiple defects are vertically positioned and evenly distributed, which means that more defective insulator may yield lower $V_{form}$. Unfortunately, it is unclear as to how many defects exist in the experimentally fabricated insulator. Moreover, the defect density strongly depends upon the process; therefore, this density should be calibrated on a case-by-case basis.

Here, we note that a serious problem regarding ReRAM is the nonuniformity in its switching operation, as mentioned earlier. From the microscopic point of view, the stochasticity in the formation of the CF induces the nonuniformity in $V_{form}$. In this regard, many efforts have been made to reduce this nonuniformity: Li et al. showed that utilizing copper ions over silver ions improves the uniformity in CB-RAM because of the faster diffusion of the former^18^, whereas Kim et al. used a cone-shaped Cu-ion source in CB-RAM^19^. Niu et al. engineered field crowding using a narrow bottom electrode, whose edge forms a field-crowding location and in turn a CF growth site, to improve the stability of a HfO_2_ ReRAM^20^. Furthermore, Lee et al. reported the improvement of uniformity upon adding metal particles to the electrolyte in CB-RAM^21^. Sun et al. demonstrated the use of an extruded electrode to induce electric-field crowding^22^, whereas Yoon et al.^23^ and Chang et al.^24^ embedded Ru and Pt nanodots in the insulator, respectively. Most of these approaches have utilized electric-field crowding via manipulating the cell geometry, although the approach details vary.

**Figure 9:**
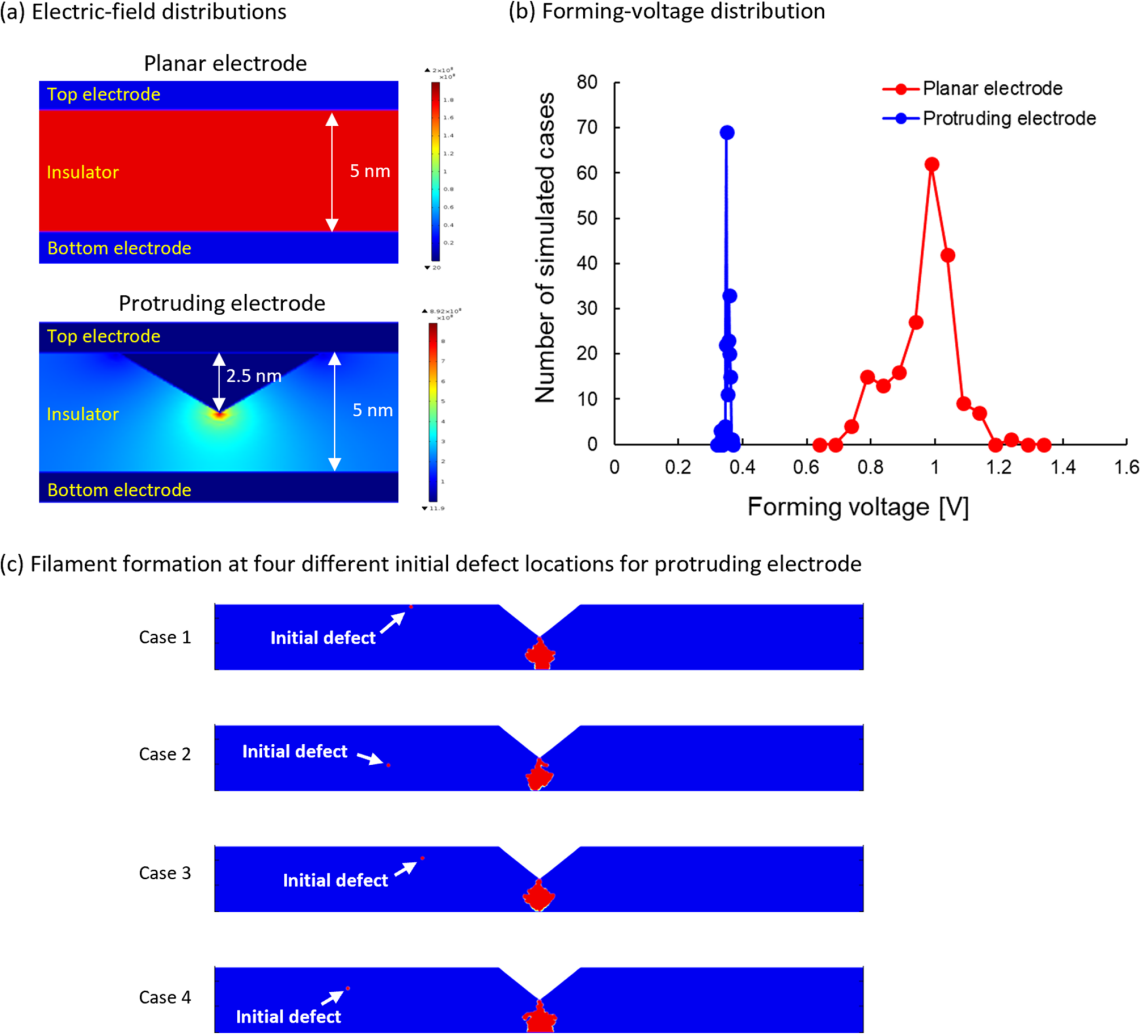
Comparison of switching uniformity between planar and protruding electrodes. (**a**) Electric field distributions of planar and protruding electrodes, (**b**) forming-voltage distributions, and (**c**) conductive filaments for four different initial defect locations for protruding electrode. In (**c**), only insulator parts are shown. The defect formation of energy of 4.5 eV was used.

In our study, we estimated the $V_{form}$ uniformity of planar and protruding electrodes using our FE simulation, which offers the advantage of incorporating the effects of the memory cell geometry; the results are presented in Fig. 9. Here, we mention that the defect formation energy of 4.5 eV was used in this simulation, which is why the $V_{form}$ value of ~ 1.0 V for this planar electrode differs from that of Fig. 6 where the thickness in both cases is 5 nm. The electric-field distributions for planar and protruding electrodes are shown in Fig. 9a. We observe that the electric field is concentrated at the tip in the case of the protruding electrode, whereas it is uniform in the case of the planar electrode. The CF can grow from the tip of the top electrode even though an initial defect exists elsewhere (not close to the tip) because the electric-field crowding is stronger at the tip than around the initial defect.

The distributions of $V_{form}$ are presented in Fig. 9b, for which data were collected from 206 and 196 simulation runs in the cases of planar and protruding electrodes, respectively. The narrower $V_{form}$-distribution of the protruding electrode implies that the filament can be formed at a predetermined location—the electric-field-crowding location. The switching-voltage difference between the electrodes arises owing to the fact that the shortest distance between the electrodes in the protruding-electrode case is half that in the planar-electrode case, which reduces the forming voltage from ~ 1 V to ~ 0.5 V (half the original value). The subsequent ~ 0.1-V reduction is explained by the electric-field crowding at the tip. The protruding electrode clearly improves $V_{form}$ uniformity, as also demonstrated in previous experiments^20,22^. If the single initial defect exists in the area directly below the vertical direction of the tip, the CF grows from the initial defect. Otherwise, the CF grows from the tip, as shown in Fig. 9c. As mentioned in the previous paragraph, this result is due to the stronger electric-field crowding at the tip.

**Figure 10:**
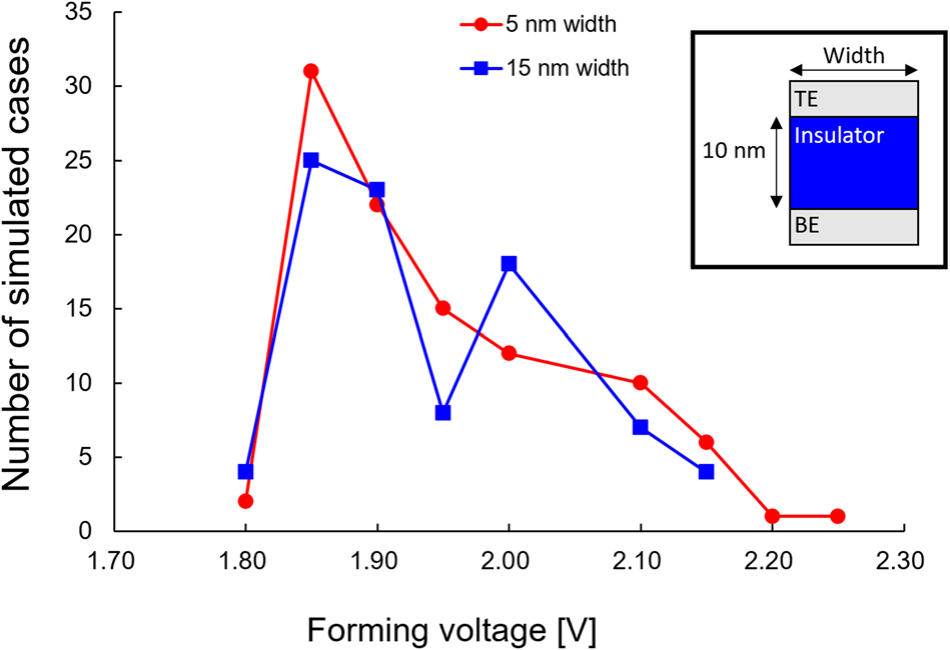
Comparison of switching uniformity for different cell widths and the same insulator thickness of 10 nm. The defect formation of energy of 4.5 eV was considered.

Next, we remark that the cell array in large-capacity ReRAM devices may be fabricated in the form of a crossbar. In this case, the memory cell has a limited width, unlike in the previous simulations that address 50-nm-width cells (Table 1). A narrower cell width implies a correspondingly lesser space available for filament formation, which is expected to yield narrower $V_{form}$-distributions. However, the distributions for the 5-nm- and 15-nm-width cells in Fig. 10 do not show any significant difference in their spread. In our study, these distributions were obtained from 100 simulation runs for each width. We speculate that this is because there is no difference in the forming voltage according to the horizontal position of the initial defect, whereas the difference in the forming voltage according to the vertical position is significant. We also note here that this result is obtained under the assumptions of perfect cell shape, completely uniform insulator composition, and the complete inertness of the electrode. In an actual situation, an electric-field-crowding-location may be formed owing to the roughness of the insulator/electrode interface and imperfect shape at the edges.

## Conclusions

In this study, we developed and performed FE simulations of the stochastic forming process in ReRAM devices. Our simulation can model the CF formation along with the corresponding current–voltage relation. On the basis of our findings, we speculate that the stochasticity in the CF formation mainly arises from the stochasticity of the initial defects including grain-boundary configurations, which induce electric-field crowding. Our results also showed that cell-geometry engineering to engineer electric-field crowding at an intended location can improve the switching uniformity. Furthermore, we demonstrated the dependence of $V_{form}$ on the properties of the active material. While our current model can reproduce the forming stochasticity in ReRAM, in the future, we plan to develop our model to simulate a full forming-reset-set cycle by incorporating more sophisticated simulations that can model thermal effects, ion and vacancy transports and their recombination, electrode material effects (contact properties), etc.

## Supplementary Information

Supplementary Information 1.
