# Supplementary material for: Investigation of switching uniformity in resistive memory via finite element simulation of conductive-filament formation
Source: Sci Rep. 2021 Jan 28;11:2447. doi: 10.1038/s41598-021-81896-z (PMC7843989; doi:10.1038/s41598-021-81896-z)
Supplement: Supplementary file 1 — Supplementary Information 1. [file 41598_2021_81896_MOESM1_ESM.docx]

Supplementary Material

**Investigation of switching uniformity in resistive memory via finite element simulation of conductive-filament formation**

Kyunghwan Min, Dongmyung Jung, and Yongwoo Kwon^*^

Department of Materials Science and Engineering, Hongik University, Seoul 04066, Korea

∗Corresponding to ykwon722@hongik.ac.kr

We provide an example m-file to simulate a single defect case. This m-file is written for the framework of Comsol Multiphysics v5.3a. Please do not run this m-file using MATLAB only. Instead, run it using Comsol LiveLink for MATLAB as guided by its documentation (https://doc.comsol.com). A 1,143-line code is shown from the next page.

% This m-file is an example simulation of a single initial defect at a

% random position that is presented in the paper titled "Investigation

% of Switching Uniformity in Resistive Memory via Finite Element Simulation

% of Conductive Filament Formation".

% Caution) Do not run this m-file directly in MATLAB. Instead, you should

% use Comsol Multiphysics with MATLAB (LiveLink for MATLAB). Please refer

% to the related documentation that can be found at the following link.

% https://doc.comsol.com/5.4/doc/com.comsol.help.llmatlab/LiveLinkForMATLABUsersGuide.pdf

% We used the COMSOL Multiphysics 5.3a. If you use a newer version,

% you may need to make appropriate changes.

% Change following paths appropriately.

% Path for "comsolinit.m" file

Path_main='C:\Program Files\COMSOL\COMSOL53a\Multiphysics\mli\startup';

% Path for storing simulation data

Path_data = 'D:\Simulation-ReRAM-v1\run7\' ;

if exist('Model_Number','var')==1

Model_Number=Model_Number+1;

else

Model_Number=1;

end

% Here Model_Number denotes a simulation run. If you perform only one

% simulation, you do not have to pay attention to this parameter. The

% distribution of forming voltage was obtained from more than 100

% simulation runs. This Model_Number variable can be used to identify

% each simulation run.

import com.comsol.model.*

import com.comsol.model.util.*

str=sprintf('model = ModelUtil.create(''Model%d'');',Model_Number);

eval(str);

model.modelPath(Path_main);

%% PARAMETERS IN MATLAB

system_unit=1e-10; % System length unit [m]

% Electric parameter

V_ramp = 1; % Voltage ramping rate [V/s]

Ic=1e-9; % Compliance current for forming

sig_LRS=3.5E4; % Electrical conductivity for LRS state [S/m]

sig_HRS=3.0E-3; % Electrical conductivity for HRS state [S/m]

area_factor=10e-9; % Area factor for 2-D simulation [m]

% Geometry parameters

% A rectangular HfO2 with top and bottom electrodes

% (Actual geometry setting is done in line 100-121)

Lx=20; % X-length of Oxide&Electrode structure [nm]

Ly=5; % Y-height of Oxide&Electrode structure [nm]

% Mesh parameters

mesh_size_max = 1; % Fine: 1 [A], coarse: 3 [A]

mesh_size_min = 0.001; % Fine: 0.001 [A], coarse: 1 [A]

% Study parameters

dt=0.1; % Minimum time step [s]

dV=0.2; % Minimum voltage step [V]

seed_number=round(rand(1)*100); % COMSOL Random seed number

cd(Path_data)

format long e;

%% PARAMETER SETTING

str=sprintf('model.param.set(''sig_LRS'', ''%d[S/m]'');',sig_LRS);

eval(str);

str=sprintf('model.param.set(''sig_HRS'', ''%d[S/m]'');',sig_HRS);

eval(str)

str=sprintf('model.param.set(''Lx'', ''%d[nm]'');',Lx);

eval(str)

str=sprintf('model.param.set(''Ly'', ''%d[nm]'');',Ly);

eval(str)

model.param.set('Ea', '5.9*e_const[V]');

model.param.set('b', ['91.8*e_const[' ...

native2unicode(hex2dec({'00' 'c5'}), 'unicode') ']']);

model.param.set('G_0', '1e30[1/(s*m^3)]');

model.param.set('k', '8.6e-5[eV/K]');

model.param.set('T', '300[K]');

model.param.set('R', ['1.4[' ...

native2unicode(hex2dec({'00' 'c5'}), 'unicode') ']']);

model.param.set('Vi', '0[V]');

str=sprintf('model.param.set(''dt'', ''%d[s]'');',dt);

eval(str);

str=sprintf('model.param.set(''t_stop'',''%d[s]'');',dt);

eval(str);

str=sprintf('model.param.set(''seed_number'', ''%d'');',seed_number);

eval(str);

model.component.create('comp1', true);

model.component('comp1').geom.create('geom1', 2);

model.result.table.create('evl2', 'Table');

model.func.create('an1', 'Analytic');

model.func('an1').set('expr', 'sig_LRS*(eta_bin==1)+sig_HRS*(eta_bin!=1)');

model.func('an1').set('args', {'eta_bin'});

model.func('an1').set('fununit', 'S/m');

model.func('an1').set('plotargs', {'eta_bin' '0' '1'});

%% MESH CREATION

model.component('comp1').mesh.create('mesh1');

%% OXIDE GEOMETRY SETTING

model.component('comp1').geom('geom1').lengthUnit( ...

[native2unicode(hex2dec({'00' 'c5'}), 'unicode') ]);

model.component('comp1').geom('geom1').create('r1', 'Rectangle');

model.component('comp1').geom('geom1').feature('r1').label('Hafnium Oxide');

model.component('comp1').geom('geom1').feature('r1').set('pos', {'-Lx/2' '0'});

model.component('comp1').geom('geom1').feature('r1').set('size', {'Lx' 'Ly'});

model.component('comp1').geom('geom1').run;

model.component('comp1').geom('geom1').run('fin');

%% TOP ELECTRODE GEOMETRY SETTING

model.component('comp1').geom('geom1').create('r2', 'Rectangle');

model.component('comp1').geom('geom1').feature('r2').set('size', {'Lx' 'Ly'});

model.component('comp1').geom('geom1').feature('r2').set('pos', {'-Lx/2' 'Ly+Ly'});

model.component('comp1').geom('geom1').runPre('fin');

model.component('comp1').geom('geom1').run;

%% BE ELECTRODE GEOMETRY SETTING

model.component('comp1').geom('geom1').create('r3', 'Rectangle');

model.component('comp1').geom('geom1').feature('r3').set('size', {'Lx' 'Ly'});

model.component('comp1').geom('geom1').feature('r3').set('pos', {'-Lx/2' 'Ly'});

model.component('comp1').geom('geom1').runPre('fin');

model.component('comp1').geom('geom1').run;

%% VARIABLE SETTING

model.component('comp1').variable.create('var1');

model.component('comp1').variable('var1').set('x1','0');

model.component('comp1').variable('var1').set('y1','Ly+Ly/2');

model.component('comp1').variable('var1').set('eta_Va', '1*((x-x1)^2+(y-y1)^2<=R^2)');

model.component('comp1').variable('var1').set('G', 'G_0*exp(-(Ea-b*ec.normE)/(k*T))');

model.component('comp1').variable('var1').set('Pc', '1-exp(-G*dt)');

model.component('comp1').variable('var1').set('eta_bin', '1*(eta_Va>=1)');

model.component('comp1').variable('var1').set('sig_m', 'an1(eta_bin)');

model.component('comp1').variable('var1').set('sigma', ...

'sig_LRS*(sig_m>=sig_LRS)+sig_HRS*(sig_m<sig_LRS)');

model.component('comp1').variable('var1').set('current_density', 'ec.normJ');

model.component('comp1').variable('var1').selection.geom('geom1', 2);

model.component('comp1').variable('var1').selection.set([1,2,3]);

model.component('comp1').variable.create('var3');

% 3rd variable indicates family of all defects

model.component('comp1').material.create('mat1', 'Common');

model.component('comp1').material('mat1').selection.set([1]);

%% RANDOM FUNCTION CREATION

model.component('comp1').func.create('rn1','Random');

model.component('comp1').func('rn1').set('nargs', 3);

model.component('comp1').func('rn1').set('mean', '0.5');

model.component('comp1').func('rn1').set('seedactive', true);

str=sprintf('model.component(''comp1'').func(''rn1'').set(''seed'', ''%d'');', ...

seed_number);

% Seed should be varied in each simulation, else random number has SAME DISTRIBUTION.

eval(str);

%% MATERIAL ASSIGNMENT

model.component('comp1').material('mat1').propertyGroup('def').set( ...

'electricconductivity', {'sigma' '0' '0' '0' 'sigma' '0' '0' '0' 'sigma'});

model.component('comp1').material('mat1').propertyGroup('def').set( ...

'relpermittivity', {'25'});

model.component('comp1').material('mat1').label('Hafnium oxide');

model.component('comp1').material('mat1').selection.set([2]);

model.component('comp1').material.create('mat2', 'Common');

model.component('comp1').material('mat2').propertyGroup('def').set( ...

'electricconductivity', {'3.5e4' '0' '0' '0' '3.5e4' '0' '0' '0' '3.5e4'});

model.component('comp1').material('mat2').propertyGroup('def').set( ...

'relpermittivity', {'1'});

model.component('comp1').material('mat2').label('ELECTRODE Metal');

model.component('comp1').material('mat2').selection.set([1,3]);

%% PHYSICS SETTING

model.component('comp1').physics.create('ec', 'ConductiveMedia', 'geom1');

model.component('comp1').physics('ec').create('pot1', 'ElectricPotential', 1);

model.component('comp1').physics('ec').feature('pot1').selection.set([7]);

model.component('comp1').physics('ec').create('gnd1', 'Ground', 1);

model.component('comp1').physics('ec').feature('gnd1').selection.set([2]);

str=sprintf('model.component(''comp1'').physics(''ec'').prop(''d'').set(''d'', ''%d[m]'');', ...

area_factor);

eval(str);

model.component('comp1').physics('ec').prop('MeshControl').set('EnableMeshControl', true);

model.component('comp1').physics('ec').feature('cucn1').set('sigma_mat', 'from_mat');

model.component('comp1').physics('ec').feature('cucn1').set('epsilonr_mat', 'from_mat');

model.component('comp1').physics('ec').feature('cucn1').set('materialType', 'solid');

model.component('comp1').physics('ec').feature('init1').set('V', '0.5*Vi');

model.component('comp1').physics('ec').feature('pot1').set('V0', 'Vi');

model.component('comp1').cpl.create('maxop1', 'Maximum');

model.component('comp1').cpl('maxop1').set('points', 'lagrange');

model.component('comp1').cpl('maxop1').selection.set([2]);

%% MESH SETTING

model.component('comp1').mesh('mesh1').create('ftri1', 'FreeTri');

model.component('comp1').mesh('mesh1').feature('ftri1').selection.geom('geom1', 2);

model.component('comp1').mesh('mesh1').feature('ftri1').selection.set([2]);

model.component('comp1').mesh('mesh1').feature('ftri1').create('size1', 'Size');

model.component('comp1').mesh('mesh1').feature('size').set('hauto', 3);

model.component('comp1').mesh('mesh1').feature('size').set('custom', 'on');

model.component('comp1').mesh('mesh1').feature('size').set('hmax', 3.0);

model.component('comp1').mesh('mesh1').feature('size').set('hmin', 2.0);

model.component('comp1').mesh('mesh1').feature('ftri1').feature('size1').set('hauto', 3);

str=sprintf( ...

'model.component(''comp1'').mesh(''mesh1'').feature(''ftri1'').feature(''size1'').set(''hmax'', %d);', ...

mesh_size_max);

eval(str);

str=sprintf( ...

'model.component(''comp1'').mesh(''mesh1'').feature(''ftri1'').feature(''size1'').set(''hmin'', %d);', ...

mesh_size_min);

eval(str);

model.component('comp1').mesh('mesh1').feature('ftri1').feature('size1').set('hgrad', 1.1);

model.component('comp1').mesh('mesh1').create('ftri2', 'FreeTri');

model.component('comp1').mesh('mesh1').feature('ftri2').selection.geom('geom1', 2);

model.component('comp1').mesh('mesh1').feature('ftri2').selection.set([1,3]);

model.component('comp1').mesh('mesh1').feature('ftri2').create('size1', 'Size');

model.component('comp1').mesh('mesh1').feature('ftri2').feature('size1').set('hauto', 3);

model.component('comp1').mesh('mesh1').feature('ftri2').feature('size1').set('hmax', 3);

model.component('comp1').mesh('mesh1').feature('ftri2').feature('size1').set('hmin', 1);

model.component('comp1').mesh('mesh1').feature('ftri2').feature('size1').set('hgrad', 1.1);

model.component('comp1').mesh('mesh1').run;

%% STUDY CREATION

model.sol.create('sol1');

model.study.create('std1');

model.study('std1').create('stat', 'Stationary');

model.sol('sol1').study('std1');

model.sol('sol1').attach('std1');

model.sol('sol1').create('st1', 'StudyStep');

model.sol('sol1').create('v1', 'Variables');

model.sol('sol1').create('s1', 'Stationary');

model.study('std1').feature('stat').set('mesh', {'geom1' 'mesh1'});

model.sol('sol1').attach('std1');

model.sol('sol1').runAll;

model.result.create('pg1', 'PlotGroup2D');

model.result('pg1').set('data', 'dset1');

model.result('pg1').create('surf1', 'Surface');

model.result('pg1').label('Electric Potential (ec)');

model.result('pg1').set('data', 'dset1');

model.result('pg1').feature('surf1').set('expr', 'ec.sigmayy');

model.result('pg1').feature('surf1').set('unit', 'S/m');

model.result('pg1').feature('surf1').set('descr', 'Electrical conductivity, yy component');

model.result('pg1').feature('surf1').set('smooth', 'internal');

model.result('pg1').feature('surf1').set('threshold', 'manual');

model.result('pg1').feature('surf1').set('resolution', 'fine');

%% Current calculation

YY=mpheval(model,'G');

[a,y0]=find(YY.p(2,:)==0); % coord of y equals to zero.

x0=YY.p(1,y0);

x0=sort(x0);

for i=1:length(x0)

if i == 1

mesh_area(1,i)=abs((x0(1)-x0(2))/2*system_unit*area_factor);

% 1st 1e-10 : unit conversion, 2nd 10e-9 : Area factor

elseif i==length(x0)

mesh_area(1,i)=abs((x0(i)-x0(i-1))/2*system_unit*area_factor);

else

mesh_area(1,i)=abs((x0(i+1)-x0(i-1))/2*system_unit*area_factor);

end

end

current_density=mpheval(model,'current_density');

current=sum(current_density.d1(1,y0).*mesh_area(1,1:length(y0)));

% Total summation of J(x)*Area

eval(str);

current_old=current;

%!!!!!!!!!!!!!!!!!!!!!!!!!!!!!!!!!!!!!!!!!!!!!!!!!!!!!!!!!!!!!!!!!!!!!!!!!!!!!!!!!!!!!!!!!!!!!!!!!!!!!!!!!!

%!!!!!!!!!!!!!!!!!!!!!!!!!!!!! AFTER RUN !!!!!!!!!!!!!!!!!!!!!!!!!!!!!!!!!!!!!!!!!!!!!!!!!!!!!!!!!!!!!!!!!!

%!!!!!!!!!!!!!!!!!!!!!!!!!!!!!!!!!!!!!!!!!!!!!!!!!!!!!!!!!!!!!!!!!!!!!!!!!!!!!!!!!!!!!!!!!!!!!!!!!!!!!!!!!!

%!!!!!!!!!!!!!!!!!!!!!!!!!!!!!!!!!!!!!!!!!!STUDY LOOP START!!!!!!!!!!!!!!!!!!!!!!!!!!!!!!!!!!!!!!!!!!!!!!!!

%!!!!!!!!!!!!!!!!!!!!!!!!!!!!!!!!!!!!!!!!!!!!!!!!!!!!!!!!!!!!!!!!!!!!!!!!!!!!!!!!!!!!!!!!!!!!!!!!!!!!!!!!!!

%% STUDY

model.physics('ec').feature('init1').set('V', '0.005');

% If unset, Initial state as an last study last step distribution.

model.variable('var1').set('sig_m', 'an1(eta_bin)');

% If unset, Initial state as an last study last step distribution.

%% STUDY NUMBERING

disp('STUDY START!!!!!');

disp('****************************************************************************************************');

disp('****************************************************************************************************');

disp('****************************************************************************************************');

if Model_Number == 1

str=sprintf('disp(''%dst STUDY START'')',Model_Number);

eval(str);

elseif Model_Number == 2

str=sprintf('disp(''%dnd STUDY START'')',Model_Number);

eval(str);

elseif Model_Number == 3

str=sprintf('disp(''%drd STUDY START'')',Model_Number);

eval(str);

else

str=sprintf('disp(''%dth STUDY START'')',Model_Number);

eval(str);

end

coords=double.empty(0,2); %No matter in case, either I.D(s) exist or not.

%% INITIAL DEFECTS ASSIGNMENT

% RANDOM NUMBER ASSIGN TO EACH MESH POINT.

rn=mpheval(model,'Pc'); % V = 0 applied, so depend on random number.

rn_matlab=rand(1,length(rn.d1)); % Distribute random number as equal number to the number of meshes.

rn.d1(:,:)=rn_matlab; % Assign the random number from MATLAB to COMSOL.

if max(rn.d1)>=0.9990

%[a,b]=find(rn.d1>=0.9994);

AAA=sort(rn.d1,'descend');

for ii = 1:length(AAA)

[a,b]=find(rn.d1(:,:)>=AAA(1,ii));

coords_i=[rn.p(1,b(ii))', rn.p(2,b(ii))']; % The coordinates of initial defect(s) in x,y respectively.

coords=coords_i;

if coords(1,2)>= Ly*10 && coords_i(1,2)<= Ly*20

break

end

end

id_x=coords(:,1);

id_y=coords(:,2);

id_x

id_y

for i = 1:length(id_x) % Coords assignment in x

str=sprintf( ...

'model.component(''comp1'').variable(''var3'').set(''initial_defect_x_%d_%d'',[''%f['' native2unicode(hex2dec({''00'' ''c5''}), ''unicode'') '']'']);', ...

i,Model_Number,coords(i,1));

eval(str);

end

for j = 1:length(id_y) % Coords assignment in y

str=sprintf( ...

'model.component(''comp1'').variable(''var3'').set(''initial_defect_y_%d_%d'',[''%f['' native2unicode(hex2dec({''00'' ''c5''}), ''unicode'') '']'']);', ...

j,Model_Number,coords(j,2));

eval(str);

end

f_i=cell(length(id_x),1);

for l=1:length(id_x) % Express all of defect from MATLAB to COMSOL

f_i{l}=strcat(sprintf('1*((x-initial_defect_x_%d_%d)^2+(y-initial_defect_y_%d_%d)^2<=R^2)', ...

l,Model_Number,l,Model_Number));

end

sumf_i=strjoin(f_i,{'+'}); % Express all of defect from MATLAB to COMSOL

str=sprintf('model.variable(''var1'').set(''eta_Va'',''%s'');',sumf_i);

% Finally, Expression is handed over to COMSOL

eval(str);

str=sprintf('disp('' # of I.D = %d'')',length(id_y));

eval(str);

else

disp('NO INITIAL DEFECTS ASSIGNED');

end

%% INITIAL STATE-DATA

% Run for data-extraction with assigned initial defects.

model.sol('sol1').runAll;

str=sprintf('model.result.export.create(''mesh_initial_%d'',''Mesh'');', ...

Model_Number);

eval(str);

str=sprintf('model.result.export(''mesh_initial_%d'').set(''data'',''dset1'');', ...

Model_Number);

eval(str);

str=sprintf('model.result.export(''mesh_initial_%d'').set(''filename'', ''%smesh_initial_%d'');', ...

Model_Number,Path_data,Model_Number);

eval(str);

str=sprintf('model.result.export(''mesh_initial_%d'').run;',Model_Number);

eval(str);

%% Element Volume Calculation

rn=mpheval(model,'Pc'); % V = 0 applied, so depend on random number.

rn_matlab=rand(1,length(rn.d1)); % Distribute random number as equal number to the number of meshes.

rn.d1(:,:)=rn_matlab; % Assign the random number from MATLAB to COMSOL.

for i=1:length(rn.t)

for a=1:3

alpha=rn.t(a,i)+1;

element_x=rn.p(1,alpha)*system_unit; % 1e-10 represents geometry scale(Angstrom)

element_y=rn.p(2,alpha)*system_unit;

if a==1

rn.element.coords(a,i)=element_x;

x1=element_x;

rn.element.coords(a+1,i)=element_y;

y1=element_y;

elseif a==2

rn.element.coords(a+1,i)=element_x;

x2=element_x;

rn.element.coords(a+2,i)=element_y;

y2=element_y;

else % a==3

rn.element.coords(a+2,i)=element_x;

x3=element_x;

rn.element.coords(a+3,i)=element_y;

y3=element_y;

end

end

rn.element.S(1,i)=0.5*abs((x1*y2+x2*y3+x3*y1)-(x2*y1+x3*y2+x1*y3)); % Matrix for area of each element

end

rn.element.coords_1=rn.element.coords(1:2,:);

rn.element.coords_2=rn.element.coords(3:4,:);

rn.element.coords_3=rn.element.coords(5:6,:);

for i = 1:length(rn.d1)

AA=[rn.p(1,i); rn.p(2,i)]*system_unit;

[Nth_column]=find(...

((rn.element.coords_1(1,:)==AA(1))&(rn.element.coords_1(2,:)==AA(2))) | ...

((rn.element.coords_2(1,:)==AA(1))&(rn.element.coords_2(2,:)==AA(2))) | ...

((rn.element.coords_3(1,:)==AA(1))&(rn.element.coords_3(2,:)==AA(2))) ); % Nth_column is meaningful.

CC=unique(Nth_column);

S_updator=0;

for j=1:length(unique(Nth_column)) % indicates the number of element sharing the node point

S_updator=S_updator+1/3*rn.element.S(1,CC(j));

end

Ve(1,i)=S_updator*area_factor;

% Ve indicates element volume for each node point //

% 10e-9 equals to area factor 10nm // Ve is consisntent in ordering with d1 component

end

% EXPORT the x,y,Ve using txt file

BB=size(length(Ve),3);

for i=1:length(Ve)

BB(i,1)=rn.p(1,i);

BB(i,2)=rn.p(2,i);

end

BB(:,3)=transpose(Ve(:,:));

% Export to text file

BB=transpose(BB);

str=sprintf('Element_Volume=fopen(''Element_Volume_%d.txt'',''w'');',Model_Number);

eval(str);

fprintf(Element_Volume,'%12e %.12e %12e\r\n',BB(:,:));

fclose(Element_Volume);

% Define the Ve distribution in COMSOL

str=sprintf('model.func.create(''int_Ve_%d'',''Interpolation'');',Model_Number);

eval(str);

str=sprintf('model.func(''int_Ve_%d'').model(''comp1'');',Model_Number);

eval(str);

str=sprintf('model.func(''int_Ve_%d'').set(''source'',''file'');',Model_Number);

eval(str);

str=sprintf('model.func(''int_Ve_%d'').set(''nargs'',''2'');',Model_Number);

eval(str);

str=sprintf('model.func(''int_Ve_%d'').set(''argunit'',[native2unicode(hex2dec({''00'' ''c5''}), ''unicode'') ]);', ...

Model_Number);

eval(str);

str=sprintf('model.func(''int_Ve_%d'').set(''fununit'',''m^3'');',Model_Number);

eval(str);

str=sprintf('model.func(''int_Ve_%d'').set(''filename'',''%sElement_Volume_%d.txt'');', ...

Model_Number,Path_data,Model_Number);

eval(str);

str=sprintf('model.func(''int_Ve_%d'').setIndex(''funcs'',''1'',0,1);',Model_Number);

eval(str);

% G variable modification by reflecting Ve

str=sprintf(...

'model.component(''comp1'').variable(''var1'').set(''G'',''G_0*int_Ve_%d(x,y)*exp(-(Ea-b*ec.normE)/(k*T))'');', ...

Model_Number);

eval(str);

clear Ve;

disp('Initial defects assigned, Time dependent study start');

%% TIME DEPENDENT STUDY CREATION

model.study.create('std2');

model.study('std2').create('time', 'Transient');

model.study('std2').feature('time').activate('ec', true);

model.study('std2').feature('time').activate('dode', true);

% Timestep-extracting

model.component('comp1').physics('ec').create('ge1', 'GlobalEquations', -1);

model.component('comp1').physics('ec').feature('ge1').setIndex('name', 'y1', 0, 0);

model.component('comp1').physics('ec').feature('ge1').setIndex('name', 'u1', 0, 0);

model.component('comp1').physics('ec').feature('ge1').setIndex('equation', 'u1-timestep', 0, 0);

% Variable-modifying

model.component('comp1').variable('var1').set('Pc', '1-exp(-G*u1[s])');

model.component('comp1').variable('var1').set('Va', 'an2(t)');

model.component('comp1').variable('var1').set('gen_time_var', 't*(ec.sigmayy==sig_LRS)');

model.component('comp1').variable('var1').set('RN_Pc','rn1(x[1/m],y[1/m],t[1/s])-Pc');

model.component('comp1').physics('ec').feature('pot1').set('V0', 'Va');

model.param.set('t0', '0');

model.param.set('t_fin','1000[s]');

% Voltage = V(t) Ramping Rate

model.func.create('an2', 'Analytic');

str=sprintf('model.func(''an2'').set(''expr'', ''1*t*%d'');',V_ramp);

eval(str)

model.func('an2').set('args', 't');

model.func('an2').set('argunit', 's');

model.func('an2').set('fununit', 'V');

% Minimum operator

model.component('comp1').cpl.create('minop1', 'Minimum');

model.component('comp1').cpl('minop1').selection.set([1]);

model.component('comp1').cpl('minop1').set('points', 'lagrange');

% Stop condition

model.sol.create('sol2');

model.sol('sol2').study('std2');

model.study('std2').feature('time').set('notlistsolnum', 1);

model.study('std2').feature('time').set('notsolnum', '1');

model.study('std2').feature('time').set('listsolnum', 1);

model.study('std2').feature('time').set('solnum', '1');

model.study('std2').feature('time').set('tlist','range(t0,1,t_fin)');

model.sol('sol2').create('st1', 'StudyStep');

model.sol('sol2').feature('st1').set('study', 'std2');

model.sol('sol2').feature('st1').set('studystep', 'time');

model.sol('sol2').create('v1', 'Variables');

model.sol('sol2').feature('v1').set('control', 'time');

model.sol('sol2').create('t1', 'Time');

model.sol('sol2').feature('t1').set('tlist', 'range(t0,1,t_fin)');

model.sol('sol2').feature('t1').set('initialstepbdfactive',true);

model.sol('sol2').feature('t1').set('maxstepbdfactive',true);

model.sol('sol2').feature('t1').set('initialstepbdf',0.1);

model.sol('sol2').feature('t1').set('maxstepbdf','1');

model.sol('sol2').feature('t1').set('plot', 'off');

model.sol('sol2').feature('t1').set('plotgroup', 'pg1');

model.sol('sol2').feature('t1').set('plotfreq', 'tout');

model.sol('sol2').feature('t1').set('probesel', 'all');

model.sol('sol2').feature('t1').set('probes', {});

model.sol('sol2').feature('t1').set('probefreq', 'tsteps');

model.sol('sol2').feature('t1').set('atolglobalvaluemethod', 'factor');

model.sol('sol2').feature('t1').set('control', 'time');

model.sol('sol2').feature('t1').create('fc1', 'FullyCoupled');

model.sol('sol2').feature('t1').feature('fc1').set('linsolver', 'dDef');

model.sol('sol2').feature('t1').feature.remove('fcDef');

model.sol('sol2').attach('std2');

model.sol('sol2').feature('t1').create('st1', 'StopCondition');

model.sol('sol2').feature('t1').feature('st1').setIndex('stopcondarr', '', 0);

model.sol('sol2').feature('t1').feature('st1').setIndex('stopcondterminateon', 'true', 0);

model.sol('sol2').feature('t1').feature('st1').setIndex('stopcondActive', true, 0);

model.sol('sol2').feature('t1').feature('st1').setIndex('stopconddesc', 'Stop expression 1', 0);

model.sol('sol2').feature('t1').feature('st1').setIndex('stopcondarr', '', 0);

model.sol('sol2').feature('t1').feature('st1').setIndex('stopcondterminateon', 'true', 0);

model.sol('sol2').feature('t1').feature('st1').setIndex('stopcondActive', true, 0);

model.sol('sol2').feature('t1').feature('st1').setIndex('stopconddesc', 'Stop expression 1', 0);

model.sol('sol2').feature('t1').feature('st1').setIndex('stopcondarr', '(comp1.minop1(comp1.RN_Pc) <=0)&&(t>t0)', 0);

model.sol('sol2').feature('t1').feature('st1').setIndex('stopcondarr', '', 1);

model.sol('sol2').feature('t1').feature('st1').setIndex('stopcondterminateon', 'true', 1);

model.sol('sol2').feature('t1').feature('st1').setIndex('stopcondActive', true, 1);

model.sol('sol2').feature('t1').feature('st1').setIndex('stopconddesc', 'Stop expression 2', 1);

model.sol('sol2').feature('t1').feature('st1').setIndex('stopcondarr', '', 1);

model.sol('sol2').feature('t1').feature('st1').setIndex('stopcondterminateon', 'true', 1);

model.sol('sol2').feature('t1').feature('st1').setIndex('stopcondActive', true, 1);

model.sol('sol2').feature('t1').feature('st1').setIndex('stopconddesc', 'Stop expression 2', 1);

model.sol('sol2').feature('t1').feature('st1').setIndex('stopcondarr', '(t>=t_stop)', 1);

model.sol('sol2').feature('t1').feature('st1').set('storestopcondsol', 'stepbefore');

% Previous solution

model.sol('sol2').feature('t1').create('ps1','PreviousSolution');

model.sol('sol2').feature('t1').feature('ps1').set('prevcomp', {'comp1_ODE1'});

voltage_matrix(1,1)=0;

time_matrix(1,1)=0;

%% STUDY-LOOP RUNNING

for j=1:1e5

if j>1

%% TIME DEPENDENT STUDY CREATION FOR EACH STEP

str=sprintf('model.study(''std2'').create(''time%d'', ''Transient'');',j);

eval(str);

str=sprintf('model.study(''std2'').feature(''time%d'').activate(''ec'', true);',j);

eval(str);

str=sprintf('model.study(''std2'').feature(''time%d'').activate(''dode'', true);',j);

eval(str);

% Stop condition

str=sprintf('model.sol.create(''sol%d'');',j+1);

eval(str);

str=sprintf('model.sol(''sol%d'').study(''std2'');',j+1);

eval(str);

str=sprintf('model.study(''std2'').feature(''time%d'').set(''tlist'',''range(t0,1,t_fin)'');',j);

eval(str);

str=sprintf('model.sol(''sol%d'').create(''st1'', ''StudyStep'');',j+1);

eval(str);

str=sprintf('model.sol(''sol%d'').feature(''st1'').set(''study'', ''std2'');',j+1);

eval(str);

str=sprintf('model.sol(''sol%d'').feature(''st1'').set(''studystep'', ''time'');',j+1);

eval(str);

str=sprintf('model.sol(''sol%d'').create(''v1'', ''Variables'');',j+1);

eval(str);

str=sprintf('model.sol(''sol%d'').feature(''v1'').set(''control'', ''time'');',j+1);

eval(str);

str=sprintf('model.sol(''sol%d'').create(''t1'', ''Time'');',j+1);

eval(str);

str=sprintf('model.sol(''sol%d'').feature(''t1'').set(''tlist'', ''range(t0,1,t_fin)'');',j+1);

eval(str);

str=sprintf('model.sol(''sol%d'').feature(''t1'').set(''plot'', ''off'');',j+1);

eval(str);

str=sprintf('model.sol(''sol%d'').feature(''t1'').set(''plotgroup'', ''pg1'');',j+1);

eval(str);

str=sprintf('model.sol(''sol%d'').feature(''t1'').set(''plotfreq'', ''tout'');',j+1);

eval(str);

str=sprintf('model.sol(''sol%d'').feature(''t1'').set(''probesel'', ''all'');',j+1);

eval(str);

str=sprintf('model.sol(''sol%d'').feature(''t1'').set(''probes'', {});',j+1);

eval(str);

str=sprintf('model.sol(''sol%d'').feature(''t1'').set(''probefreq'', ''tsteps'');',j+1);

eval(str);

str=sprintf('model.sol(''sol%d'').feature(''t1'').set(''atolglobalvaluemethod'', ''factor'');',j+1);

eval(str);

str=sprintf('model.sol(''sol%d'').feature(''t1'').set(''control'', ''time'');',j+1);

eval(str);

str=sprintf('model.sol(''sol%d'').feature(''t1'').create(''fc1'', ''FullyCoupled'');',j+1);

eval(str);

str=sprintf('model.sol(''sol%d'').feature(''t1'').feature(''fc1'').set(''linsolver'', ''dDef'');',j+1);

eval(str);

str=sprintf('model.sol(''sol%d'').feature(''t1'').feature.remove(''fcDef'');',j+1);

eval(str);

str=sprintf('model.sol(''sol%d'').attach(''std2'');',j+1);

eval(str);

str=sprintf('model.sol(''sol%d'').feature(''t1'').create(''st1'', ''StopCondition'');',j+1);

eval(str);

str=sprintf('model.sol(''sol%d'').feature(''t1'').feature(''st1'').setIndex(''stopcondarr'', '''', 0);',j+1);

eval(str);

str=sprintf('model.sol(''sol%d'').feature(''t1'').feature(''st1'').setIndex(''stopcondterminateon'', ''true'', 0);',j+1);

eval(str);

str=sprintf('model.sol(''sol%d'').feature(''t1'').feature(''st1'').setIndex(''stopcondActive'', true, 0);',j+1);

eval(str);

str=sprintf('model.sol(''sol%d'').feature(''t1'').feature(''st1'').setIndex(''stopconddesc'', ''Stop expression 1'', 0);',j+1);

eval(str);

str=sprintf('model.sol(''sol%d'').feature(''t1'').feature(''st1'').setIndex(''stopcondarr'', '''', 0);',j+1);

eval(str);

str=sprintf('model.sol(''sol%d'').feature(''t1'').feature(''st1'').setIndex(''stopcondterminateon'', ''true'', 0);',j+1);

eval(str);

str=sprintf('model.sol(''sol%d'').feature(''t1'').feature(''st1'').setIndex(''stopcondActive'', true, 0);',j+1);

eval(str);

str=sprintf('model.sol(''sol%d'').feature(''t1'').feature(''st1'').setIndex(''stopconddesc'', ''Stop expression 1'', 0);',j+1);

eval(str);

str=sprintf('model.sol(''sol%d'').feature(''t1'').feature(''st1'').setIndex(''stopcondarr'', ''(comp1.minop1(comp1.RN_Pc)<=0)&&(t>t0)'', 0);',j+1);

eval(str);

str=sprintf('model.sol(''sol%d'').feature(''t1'').feature(''st1'').setIndex(''stopcondarr'', '''', 1);',j+1);

eval(str);

str=sprintf('model.sol(''sol%d'').feature(''t1'').feature(''st1'').setIndex(''stopcondterminateon'', ''true'', 1);',j+1);

eval(str);

str=sprintf('model.sol(''sol%d'').feature(''t1'').feature(''st1'').setIndex(''stopcondActive'', true, 1);',j+1);

eval(str);

str=sprintf('model.sol(''sol%d'').feature(''t1'').feature(''st1'').setIndex(''stopconddesc'', ''Stop expression 2'', 1);',j+1);

eval(str);

str=sprintf('model.sol(''sol%d'').feature(''t1'').feature(''st1'').setIndex(''stopcondarr'', '''', 1);',j+1);

eval(str);

str=sprintf('model.sol(''sol%d'').feature(''t1'').feature(''st1'').setIndex(''stopcondterminateon'', ''true'', 1);',j+1);

eval(str);

str=sprintf('model.sol(''sol%d'').feature(''t1'').feature(''st1'').setIndex(''stopcondActive'', true, 1);',j+1);

eval(str);

str=sprintf('model.sol(''sol%d'').feature(''t1'').feature(''st1'').setIndex(''stopconddesc'', ''Stop expression 2'', 1);',j+1);

eval(str);

str=sprintf('model.sol(''sol%d'').feature(''t1'').feature(''st1'').setIndex(''stopcondarr'', ''(t>=t_stop)'', 1);',j+1);

eval(str);

str=sprintf('model.sol(''sol%d'').feature(''t1'').feature(''st1'').set(''storestopcondsol'', ''stepafter'');',j+1);

eval(str);

end

str=sprintf('model.sol(''sol%d'').runAll;',j+1);

eval(str);

%% When solver stopped

str=sprintf('gen_time=mpheval(model,''gen_time_var'',''dataset'',''dset%d'');',j+1);

eval(str);

gen_time=max(max(gen_time.d1));

str=sprintf('model.param.set(''gen_time'', ''%0.15f'');',gen_time);

eval(str);

str=sprintf('Va=mpheval(model,''Va'',''dataset'',''dset%d'',''t'',%0.15f);',j+1,gen_time);

eval(str);

Va=max(max(Va.d1));

voltage_matrix(j+1,1)=Va;

time_matrix(j+1,1)=gen_time;

%% Defect generation

str=sprintf('RN_Pc=mpheval(model,''RN_Pc'',''dataset'',''dset%d'',''t'',%0.15f,''smooth'',''none'');',j+1,gen_time);

eval(str);

str=sprintf('sigma=mpheval(model,''sigma'',''dataset'',''dset%d'',''t'',%0.15f,''smooth'',''none'');',j+1,gen_time);

eval(str);

str=sprintf('disp(''V = %0.5f'');',Va);

eval(str);

str=sprintf('disp(''t= %0.5f'');',gen_time);

eval(str);

A=RN_Pc.d1(:,:)<=0;

[a,b]=find(A==1);

o=0;

p=0;

if isempty(b)==1

disp('Current check or Overlapped defects');

elseif ~isempty(b)

str=sprintf('disp(''at t = %0.5f, Defect-generation occurs'');',gen_time);

eval(str);

str=sprintf('disp(''at V = %0.5f, Defect-generation occurs'');',Va);

eval(str);

if isempty(coords)==0 % Case in initial defects exist

coords=[coords; RN_Pc.p(1,b)', RN_Pc.p(2,b)'];

str=sprintf('disp(''The number of generated defect NON-OVERLAPPED = %d'');',length(b));

eval(str);

% Make a circular shape for each circle

for o=o+1:length(b)+o % for x coordinates

str=sprintf( ...

'model.component(''comp1'').variable(''var3'').set(''defect_x_%d_%d'',[''%f['' native2unicode(hex2dec({''00'' ''c5''}), ''unicode'') '']'']);', ...

o,Model_Number,coords(o,1));

eval(str);

end

for p=p+1:length(b)+p % for y coordinates

str=sprintf( ...

'model.component(''comp1'').variable(''var3'').set(''defect_y_%d_%d'',[''%f['' native2unicode(hex2dec({''00'' ''c5''}), ''unicode'') '']'']);', ...

p,Model_Number,coords(p,2));

eval(str);

end

o=o-length(b);

% Assign newly-generated defects as a variable each step.

for k=o+1:o+length(b) %

f{k}=strcat(sprintf('1*((x-defect_x_%d_%d)^2+(y-defect_y_%d_%d)^2<=R^2)',k,Model_Number,k,Model_Number));

% Only for newly-generated defects in this step.

end

sumf=strjoin(f(o+1:o+length(b)),{'+'});

sumf;

str=sprintf('model.variable(''var1'').set(''eta_Va'',''%s'');',sumf);

eval(str);

o=k;

str=sprintf('model.sol(''sol%d'').runAll;',j+1);

eval(str);

else % isempty(coords)==1

coords=[RN_Pc.p(1,b)',RN_Pc.p(2,b)']; % Case in no initial deefects

str=sprintf('disp(''The number of generated defects NON-OVERLAPPED = %d'');',length(b));

eval(str);

% Make a circular shape for each circle

for o=o+1:length(b)+o % for x coordinates

str=sprintf( ...

'model.component(''comp1'').variable(''var3'').set(''defect_x_%d_%d'',[''%f['' native2unicode(hex2dec({''00'' ''c5''}), ''unicode'') '']'']);', ...

o,Model_Number,coords(o,1));

eval(str);

end

for p=p+1:length(b)+p % for y coordinates

str=sprintf( ...

'model.component(''comp1'').variable(''var3'').set(''defect_y_%d_%d'',[''%f['' native2unicode(hex2dec({''00'' ''c5''}), ''unicode'') '']'']);', ...

p,Model_Number,coords(p,2));

eval(str);

end

o=o-length(b);

% Assign newly-generated defects as a variable each step.

for k=o+1:o+length(b) %

f{k}=strcat(sprintf('1*((x-defect_x_%d_%d)^2+(y-defect_y_%d_%d)^2<=R^2)',k,Model_Number,k,Model_Number));

% Only for newly-generated defects in this step.

end

sumf=strjoin(f(o+1:o+length(b)),{'+'});

sumf;

str=sprintf('model.variable(''var1'').set(''eta_Va'',''%s'');',sumf);

eval(str);

o=k;

str=sprintf('model.sol(''sol%d'').runAll;',j+1);

eval(str);

end

end

if j==1

model.result.create('pg2', 'PlotGroup2D');

model.result('pg2').set('data', 'dset2');

model.result('pg2').create('surf1', 'Surface');

model.result('pg2').label('Electric Potential Time dependent (ec)');

model.result('pg2').set('data', 'dset2');

model.result('pg2').feature('surf1').set('expr', 'ec.sigmayy');

model.result('pg2').feature('surf1').set('unit', 'S/m');

model.result('pg2').feature('surf1').set('descr', 'Electrical conductivity, yy component');

model.result('pg2').feature('surf1').set('smooth', 'internal');

model.result('pg2').feature('surf1').set('threshold', 'manual');

model.result('pg2').feature('surf1').set('resolution', 'fine');

str=sprintf('model.result(''pg2'').feature(''surf1'').set(''interp'', [%0.15f]);',gen_time);

eval(str);

end

%DATA-EXPORTING

% SIGMA

if j==1

str=sprintf('model.result.export.create(''data_sig_%d'',''Data'');',Model_Number);

eval(str);

end

str=sprintf('model.result.export(''data_sig_%d'').set(''data'',''dset%d'');',Model_Number,j+1);

eval(str);

str=sprintf('model.result.export(''data_sig_%d'').setIndex(''looplevelinput'', ''last'', 0);',Model_Number);

eval(str);

str=sprintf('model.result.export(''data_sig_%d'').set(''expr'',{''ec.sigmayy''});',Model_Number);

eval(str);

str=sprintf('model.result.export(''data_sig_%d'').set(''descr'', {''Electrical conductivity''});',Model_Number);

eval(str);

str=sprintf('model.result.export(''data_sig_%d'').set(''unit'', {''S/m''});',Model_Number);

eval(str);

str=sprintf('model.result.export(''data_sig_%d'').set(''filename'',''%ssig%d_%d.txt'');',Model_Number,Path_data,gen_time,Model_Number);

eval(str);

str=sprintf('model.result.export(''data_sig_%d'').run;',Model_Number);

eval(str);

if j==1

str=sprintf('model.func.create(''int_sig_%d'',''Interpolation'');',Model_Number);

eval(str);

str=sprintf('model.func(''int_sig_%d'').model(''comp1'');',Model_Number);

eval(str);

str=sprintf('model.func(''int_sig_%d'').set(''source'',''file'');',Model_Number);

eval(str);

str=sprintf('model.func(''int_sig_%d'').set(''nargs'',''2'');',Model_Number);

eval(str);

str=sprintf('model.func(''int_sig_%d'').set(''argunit'',[native2unicode(hex2dec({''00'' ''c5''}), ''unicode'') ]);',Model_Number);

eval(str);

str=sprintf('model.func(''int_sig_%d'').set(''fununit'',''S/m'');',Model_Number);

eval(str);

end

str=sprintf('model.func(''int_sig_%d'').set(''filename'',''%ssig%d_%d.txt'');',Model_Number,Path_data,gen_time,Model_Number);

eval(str);

str=sprintf('model.func(''int_sig_%d'').setIndex(''funcs'',''1'',0,1);',Model_Number);

eval(str);

% POTENTIAL

if j==1

str=sprintf('model.result.export.create(''data_V_%d'',''Data'');',Model_Number);

eval(str);

end

str=sprintf('model.result.export(''data_V_%d'').set(''data'', ''dset%d'');',Model_Number,j+1);

eval(str);

str=sprintf('model.result.export(''data_V_%d'').setIndex(''looplevelinput'', ''last'', 0);',Model_Number);

eval(str);

str=sprintf('model.result.export(''data_V_%d'').set(''expr'',{''V''});',Model_Number);

eval(str);

str=sprintf('model.result.export(''data_V_%d'').set(''descr'', {''Electric potential''});',Model_Number);

eval(str);

str=sprintf('model.result.export(''data_V_%d'').set(''unit'', {''V''});',Model_Number);

eval(str);

str=sprintf('model.result.export(''data_V_%d'').set(''filename'',''%sPotential%d_%d.txt'');',Model_Number,Path_data,gen_time,Model_Number);

eval(str);

str=sprintf('model.result.export(''data_V_%d'').run;',Model_Number);

eval(str);

if j==1

str=sprintf('model.func.create(''int_V_%d'',''Interpolation'');',Model_Number);

eval(str);

str=sprintf('model.func(''int_V_%d'').model(''comp1'');',Model_Number);

eval(str);

str=sprintf('model.func(''int_V_%d'').set(''source'', ''file'');',Model_Number);

eval(str);

str=sprintf('model.func(''int_V_%d'').set(''nargs'',''2'');',Model_Number);

eval(str);

str=sprintf('model.func(''int_V_%d'').set(''argunit'',[native2unicode(hex2dec({''00'' ''c5''}), ''unicode'') ]);',Model_Number);

eval(str);

str=sprintf('model.func(''int_V_%d'').set(''fununit'',''V'');',Model_Number);

eval(str);

end

str=sprintf('model.func(''int_V_%d'').set(''filename'', ''%sPotential%d_%d.txt'');',Model_Number,Path_data,gen_time,Model_Number);

eval(str);

% PROBABILITY

if j==1

str=sprintf('model.result.export.create(''data_Pc_%d'',''Data'');',Model_Number);

eval(str);

end

str=sprintf('model.result.export(''data_Pc_%d'').set(''data'', ''dset%d'');',Model_Number,j+1);

eval(str);

str=sprintf('model.result.export(''data_Pc_%d'').setIndex(''looplevelinput'', ''last'', 0);',Model_Number);

eval(str);

str=sprintf('model.result.export(''data_Pc_%d'').set(''expr'',{''Pc''});',Model_Number);

eval(str);

str=sprintf('model.result.export(''data_Pc_%d'').set(''descr'', {''Probability''});',Model_Number);

eval(str);

str=sprintf('model.result.export(''data_Pc_%d'').set(''filename'',''%sPc%d_%d.txt'');',Model_Number,Path_data,gen_time,Model_Number);

eval(str);

str=sprintf('model.result.export(''data_Pc_%d'').run;',Model_Number);

eval(str);

if j==1

str=sprintf('model.func.create(''int_Pc_%d'',''Interpolation'');',Model_Number);

eval(str);

str=sprintf('model.func(''int_Pc_%d'').model(''comp1'');',Model_Number);

eval(str);

str=sprintf('model.func(''int_Pc_%d'').set(''source'', ''file'');',Model_Number);

eval(str);

str=sprintf('model.func(''int_Pc_%d'').set(''nargs'',''2'');',Model_Number);

eval(str);

str=sprintf('model.func(''int_Pc_%d'').set(''argunit'',[native2unicode(hex2dec({''00'' ''c5''}), ''unicode'') ]);',Model_Number);

eval(str);

end

str=sprintf('model.func(''int_Pc_%d'').set(''filename'', ''%sPc%d_%d.txt'');',Model_Number,Path_data,gen_time,Model_Number);

eval(str);

model.result('pg2').run;

% Set up as an initial state.

str=sprintf('model.physics(''ec'').feature(''init1'').set(''V'', ''int_V_%d(x,y)'');',Model_Number);

eval(str);

if j==1

str=sprintf('model.result.export.create(''mesh_%d'',''Mesh'');',Model_Number);

eval(str);

end

str=sprintf('model.result.export(''mesh_%d'').set(''data'',''dset%d'');',Model_Number,j+1);

eval(str);

str=sprintf('model.result.export(''mesh_%d'').set(''filename'', ''%smesh%d_%d'');',Model_Number,Path_data,j,Model_Number);

eval(str);

str=sprintf('model.result.export(''mesh_%d'').run;',Model_Number);

eval(str);

% Parameter-modifying

str=sprintf('model.param.set(''t0'',%d);',gen_time);

eval(str);

%RANDOM NUMBER ASSIGN TO EACH MESH POINT.

str=sprintf('rn=mpheval(model,''Pc'',''dataset'',''dset2'',''t'',%0.15f);',gen_time);

eval(str);

rn_matlab=rand(1,length(rn.d1)); % Distribute the random number as same as the number of meshes.

rn.d1(:,:)=rn_matlab; % Assign the random number from MATLAB to COMSOL.

% Element Volume Calculation

for i=1:length(rn.t)

for a=1:3

alpha=rn.t(a,i)+1;

element_x=rn.p(1,alpha)*system_unit; % 1e-10 represents geometry scale(Angstrom)

element_y=rn.p(2,alpha)*system_unit;

if a==1

rn.element.coords(a,i)=element_x;

x1=element_x;

rn.element.coords(a+1,i)=element_y;

y1=element_y;

elseif a==2

rn.element.coords(a+1,i)=element_x;

x2=element_x;

rn.element.coords(a+2,i)=element_y;

y2=element_y;

else % a==3

rn.element.coords(a+2,i)=element_x;

x3=element_x;

rn.element.coords(a+3,i)=element_y;

y3=element_y;

end

end

rn.element.S(1,i)=0.5*abs((x1*y2+x2*y3+x3*y1)-(x2*y1+x3*y2+x1*y3)); % Matrix for area of each element

end

rn.element.coords_1=rn.element.coords(1:2,:);

rn.element.coords_2=rn.element.coords(3:4,:);

rn.element.coords_3=rn.element.coords(5:6,:);

for i = 1:length(rn.d1)

AA=[rn.p(1,i); rn.p(2,i)]*system_unit;

[Nth_column]=find(...

((rn.element.coords_1(1,:)==AA(1))&(rn.element.coords_1(2,:)==AA(2))) | ...

((rn.element.coords_2(1,:)==AA(1))&(rn.element.coords_2(2,:)==AA(2))) | ...

((rn.element.coords_3(1,:)==AA(1))&(rn.element.coords_3(2,:)==AA(2))) ); % Nth_column is meaningful.

CC=unique(Nth_column);

S_updator=0;

for AA=1:length(unique(Nth_column)) % indicates the number of element sharing the node point

S_updator=S_updator+1/3*rn.element.S(1,CC(AA));

end

Ve(1,i)=S_updator*area_factor;

% Ve indicates element volume for each node point //

% 10e-9 equals to area factor 10nm // Ve is consisntent in ordering with d1 component

end

% EXPORT the x,y,Ve using txt file

BB=size(length(Ve),3);

for i=1:length(Ve)

BB(i,1)=rn.p(1,i); % x coords

BB(i,2)=rn.p(2,i); % y coords

end

BB(:,3)=transpose(Ve(:,:)); % Element Volume

% Export to text file

BB=transpose(BB);

str=sprintf('Element_Volume=fopen(''Element_Volume_%d.txt'',''w'');',Model_Number);

eval(str);

fprintf(Element_Volume,'%12e %.12e %12e\r\n',BB(:,:));

fclose(Element_Volume);

str=sprintf('model.func(''int_Ve_%d'').set(''filename'',''%sElement_Volume_%d.txt'');',Model_Number,Path_data,Model_Number);

eval(str);

str=sprintf('model.func(''int_Ve_%d'').setIndex(''funcs'',''1'',0,1);',Model_Number);

eval(str);

clear Ve;

%% Current calculation

str=sprintf('ZZ=mpheval(model,''G'',''dataset'',''dset%d'',''t'',%0.15f);',j+1,gen_time);

eval(str);

[z,y0]=find(ZZ.p(2,:)==0); % at y==0

x0=ZZ.p(1,y0); % coords of x in y==0

x0=sort(x0);

for i=1:length(x0)

if i == 1

mesh_area(1,i)=abs((x0(1)-x0(2))/2*system_unit*area_factor); % 1st 1e-10 : unit conversion, 2nd 10e-9 : area factor 10[nm]

elseif i==length(x0)

mesh_area(1,i)=abs((x0(i)-x0(i-1))/2*system_unit*area_factor);

else

mesh_area(1,i)=abs((x0(i+1)-x0(i-1))/2*system_unit*area_factor);

end

end

str=sprintf('current_density_%d=mpheval(model,''current_density'',''dataset'',''dset%d'',''t'',%0.15f);',Model_Number,j+1,gen_time);

eval(str);

str=sprintf('current_%d=sum(current_density_%d.d1(1,y0).*mesh_area(1,1:length(y0)));',Model_Number,Model_Number); %Total summation of J(x)*A

eval(str);

str1=sprintf('current_%d',Model_Number);

str=sprintf('disp(''Current = %d'');',eval(str1));

eval(str);

disp('*************************************************************************************');

disp('*************************************************************************************');

str=sprintf('current_matrix=[current_old;current_%d];',Model_Number);

eval(str);

str=sprintf('model.param.set(''V_stop'',''%d[V]'');',Va+dV);

eval(str);

str=sprintf('model.param.set(''t_stop'',''%d[s]'');',gen_time+dt);

eval(str);

current_old=current_matrix;

if eval(str1) > Ic

str=sprintf('disp(''Current has reached Ic at V = %d '');',Va);

eval(str);

A_TOTAL=[transpose(time_matrix); transpose(voltage_matrix); transpose(current_matrix(:,1))];

str=sprintf('CURRENT_MATRIX=fopen(''Current_%d.txt'',''w'');',Model_Number);

eval(str);

fprintf(CURRENT_MATRIX,'%12s\r\n','Current');

fprintf(CURRENT_MATRIX,'%.12e %.12e %.12e\r\n',A_TOTAL);

fclose(CURRENT_MATRIX);

str=sprintf('disp(''Seed number = %d'');',seed_number);

eval(str);

break

end

end

cd ('../')

clearvars -except Model_Number;
